# Genome-based classification of *Paraniabella aurantiaca* gen. nov., sp. nov., isolated from soil and taxonomic reclassification of five species within the genus *Niabella*

**DOI:** 10.71150/jm.2505005

**Published:** 2025-10-31

**Authors:** Yong-Seok Kim, Yerang Yang, Miryung Kim, Do-Hoon Lee, Chang-Jun Cha

**Affiliations:** Department of Systems Biotechnology, Chung-Ang University, Anseong 17546, Republic of Korea

**Keywords:** *Paraniabella*, *Paraniabella aurantiaca*, genome, reclassification, OGRI

## Abstract

A Gram-stain-negative, aerobic, non-motile, rod-shaped, and orange-pigmented bacterium, designated CJ426^T^, was isolated from ginseng soil in Anseong, Korea. Strain CJ426^T^ grew optimally on Reasoner’s 2A agar at 30°C and pH 7.0 in the absence of NaCl. Phylogenetic analysis based on the 16S rRNA gene sequence revealed that strain CJ426^T^ belonged to the family *Chitinophagaceae* and had the highest sequence similarity with *Niabella hibiscisoli* KACC 18857^T^ (98.7%). The 16S rRNA gene sequence similarities with other members of the genus *Niabella* ranged from 92.3% to 98.1%. Phylogenomic analyses and overall genomic relatedness indices, including average nucleotide identity, average amino acid identity, and the percentage of conserved proteins values, supported the classification of strain CJ426^T^ as a representative of a novel genus within the family *Chitinophagaceae*. Furthermore, genome-based analyses suggested that five members of the genus *Niabella*, including *N. aquatica*, *N. defluvii*, *N. ginsengisoli*, *N. hibiscisoli*, and, *N. yanshanensis*, should be separated from other *Niabella* species and be assigned as a novel genus. The major isoprenoid quinone of strain CJ426^T^ was menaquinone-7 (MK-7). The predominant polar lipids were phosphatidylethanolamine and six unidentified aminolipids. The major fatty acids were iso-C_15:0_, iso-C_15:1_ G, and iso-C_17:0_ 3-OH. The genome of strain CJ426^T^ was 6.3 Mbp in size, consisting of three contigs, with a G + C content of 41.9%. Based on a polyphasic taxonomic approach, strain CJ426^T^ represents a novel genus and species within the family *Chitinophagaceae*, for which the name *Paraniabella aurantiaca* gen. nov., sp. nov. is proposed. The type strain is CJ426^T^ (= KACC 23908^T^ = JCM 37728^T^).

## Introduction

The family *Chitinophagaceae*, belonging to the order *Chitinophagales* within the phylum *Bacteroidota*, was initially proposed by Kämpfer et al. (2011) and has been reported in a wide range of habitats, including soil, fresh water, marine, air, plants, humans, and hot springs. The members of the family are characterized as Gram-stain-negative, usually non-motile, rod-shaped, and either aerobic or facultatively anaerobic. At the time of the current writing, the List of Prokaryotic Names with Standing in Nomenclature (LPSN) encompassed 56 validly published names within the family *Chitinophagaceae* (Parte et al., 2020).

The genus *Niabella* was first proposed by Kim et al. (2007). Phylogenetically, the genus *Niabella* belongs to the family *Chitinophagaceae* in the class *Chitinophagia*. Currently, LPSN listed 14 validly published names under the genus *Niabella* (Parte et al., 2020). Members of the genus *Niabella* have been isolated from diverse sources, including lake water (Siddiqi and Im, 2016), influent water (Min et al., 2024), wetlands (Guo et al., 2022), soil samples (Dahal and Kim, 2016; Dai et al., 2011; Kim et al., 2007; Lin et al., 2022; Ngo et al., 2017; Wang et al., 2009; Weon et al., 2008, 2009), compost (Yi et al., 2015), and medicinal leeches (*Hirudo verbena*) (Glaeser et al., 2013). The genus is characterized as Gram-stain-negative, short rod-shaped, aerobic, non-flagellated, non-spore-forming, and flexirubin-pigment-producing. The major respiratory quinone is menaquinone-7 (MK-7), and the major fatty acids include iso-C_15:0_, iso-C_15:1_ G, and iso-C_17:0_ 3-OH.

In this study, a novel bacterium, designated strain CJ426^T^, was isolated from ginseng soil and characterized using a polyphasic taxonomic approach in comparison with related type strains. Based on polyphasic taxonomic analyses, strain CJ426^T^ is proposed as a novel species and genus, for which the name *Paraniabella aurantiaca* gen. nov., sp. nov. is proposed. Furthermore, genome-based phylogenetic analyses suggest that the closely related phylogenetic neighbours *N. aquatica*, *N. defluvii*, *N. ginsengisoli*, *N. hibiscisoli*, and *N. yanshanensis* be reclassified into this novel genus.  

## Materials and Methods

### Isolation of bacterial strain and culture conditions

A novel bacterial strain was isolated from ginseng soil in Anseong, Korea (37°02'24.8"N 127°21'58.6"E). The ginseng soil samples (approximately 1–2 g) were suspended in 50 ml of R2A broth (BD) and incubated at 30℃ for 48 h with shaking (200 rpm). The enriched culture was then serially diluted, spread onto R2A agar plates, and incubated at 30℃ for 48 h. Several single colonies were picked and purified by repeated streaking on fresh R2A agar plates. Among them, strain CJ426^T^ was cultivated in R2A broth (BD) at 30℃ for two days and preserved in a 30% (w/v) glycerol suspension at –80℃.

### 16S rRNA gene-based phylogenetic analysis

The 16S rRNA gene was amplified from a single colony via PCR using AccuPower PCR Premix (Bioneer) and primers pBact27F and pUniv1492R (Frank et al., 2008). Sequencing of the 16S rRNA gene was performed at Biofact (Korea) on an automated DNA analyzer (ABI 3730XL; Applied Biosystems) using primers 27F, 1492R, 518F, and 805R to generate a consensus sequence (Baker et al., 2003). The partial 16S rRNA gene sequence of strain CJ426^T^ was compared with those of related type strains using the EzBioCloud server (Chalita et al., 2024). Multiple sequence alignments were performed using MUSCLE (Edgar, 2004), and phylogenetic trees were constructed using the neighbour-joining (NJ), maximum-likelihood (ML), and maximum-parsimony (MP) methods in MEGA 11 software (Tamura et al., 2021). The NJ phylogeny was constructed using the Jukes–Cantor model (Jukes and Cantor, 1969). For the ML phylogeny, the best substitution model was determined based on the Bayesian Information Criterion using the model test option in MEGA 11, and the Kimura 2-parameter model was applied to construct the ML phylogeny. The MP phylogeny was constructed using the pairwise deletion option. Bootstrap analysis with 1000 replicates was employed to evaluate tree topologies (Felsenstein, 1985).

### Genomic DNA extraction, whole genome sequencing, and de novo assembly

Genomic DNA was extracted using the DNeasy Blood & Tissue Kits (QIAGEN, Germany) following the manufacturer’s protocols. The whole genome sequencing of strain CJ426^T^ was conducted at CJ Bioscience, Inc. (Korea) using the PacBio sequencing platform. The genomic DNA library for sequencing was prepared using the SMRTbell^®^ Express Template Preparation Kit (PacBio, Cat No, 100-938-900). Genome assembly was performed with SMRTLink v13.1.0 using the Microbial Assembly protocol (Pacific Biosciences, USA). Contigs were rearranged to start at the dnaA/repA replication origin using Circlator v1.5.5 (Hunt et al., 2015). Reconstruction and typing of bacterial plasmid contigs from draft genome assembly data was used MOB-suite (Robertson and Nash, 2018). The assembled genome was evaluated using BUSCO v5.8.0 with single-copy gene datasets of *Bacteroidota* lineages v5 (Manni et al., 2021) and CheckM v1.2.3 with the marker lineage “p__Bacteroidetes” following to the default parameters. The assembled genome was visualized using Genovi v0.2.16 (Cumsille et al., 2023).

### Genomic and phylogenomic analyses

The genomic analysis adhered to the minimal standards for genome data proposed by Chun et al. (2018). Digital DNA-DNA hybridization (dDDH) values were calculated using the Genome-to-Genome Distance Calculator (GGDC 3.0) (Meier-Kolthoff et al., 2022) with formula 2. Average nucleotide identity (ANI) values were obtained using the OrthoANI pipeline (Lee et al., 2016). In accordance with the updated minimal standards for genome data at the genus level proposed by Riesco and Trujillo (2024), average amino acid identity (AAI) values were determined using the EzAAI pipeline (Kim et al., 2021b), and the percentage of conserved proteins (POCP) for genus-level classification was calculated according to Qin et al. (2014). Reference genome sequences for comparison with strain CJ426^T^ were sourced from the National Center for Biotechnology Information (NCBI) datasets (O'Leary et al., 2024) and the Type Strains Genome database (gcTYPE) (Fan et al., 2024).

Protein-coding genes, rRNA, tRNA, and tmRNA genes were predicted using Prodigal (Hyatt et al., 2010), Infernal (Nawrocki and Eddy, 2013) with bacterial covariance models based on the Pfam database (Kalvari et al., 2021; Schwengers et al., 2021), tRNAscan-SE 2.0 (Chan et al., 2021), and Aragorn (Laslett and Canback, 2004), respectively. Functional annotation was performed using Bakta v1.9.4 (Schwengers et al., 2021), incorporating data from three UniProt Reference Cluster databases (UniProt, 2021) and ten additional databases. The functional metabolic pathways of type strains in the genus *Niabella* were reconstructed using the KEGG database within METABOLIC v4.0 software (Zhou et al., 2022). Carbohydrate-active enzymes and proteolytic enzymes were annotated with HMMER v3.4 (Finn et al., 2011) using the CAZy database implemented in the dbCAN2 server (Zheng et al., 2023) and the MEROPS database (Rawlings et al., 2017), respectively. Antibiotic resistance genes (ARGs) were identified using the NCBI Antimicrobial Resistance Gene Finder (Feldgarden et al., 2021). Secondary metabolite biosynthetic gene clusters (BGCs) were identified using antiSMASH v7.0 (Blin et al., 2024).

The up-to-date bacterial core-genes 2 (UBCG2) phylogenomic pipeline (Kim et al., 2021a) was used to generate a concatenated alignment of 81 UBCG sequences, and a phylogenomic tree was constructed using IQ-TREE v2.3.5 (Minh et al., 2020) with the transitional (TIM3) substitution model, empirical base frequencies, invariable sites, and four gamma categories. The constructed phylogenomic tree was visualized using MEGA 11 software. The phylogeny produced using UBCGs was compared with trees generated by other methods, including a phylogenomic tree based on 290 concatenated core-gene alignment sequences extracted by Panaroo v1.5.1 (Tonkin-Hill et al., 2020). Alternative trees were also constructed using IQ-TREE v2.3.5 with the general time reversible substitution model, empirical base frequencies, invariable sites, and four gamma categories.

The pan-genomes of type strains in the genus *Niabella* were calculated using Panaroo v1.5.1, with Bakta-annotated genomes as input. A protein sequence identity threshold of 90% (--core_threshold 0.90) and a length difference cutoff of 98% (--len_dif_percent 0.98) were applied in strict mode (--clean-mode strict). The protein family sequence identity threshold was set to 50% (-f 0.5). The assembled genome was compared with the reference genomes of related type strains through whole-genome alignment using NUCmer v3.23 (Kurtz et al., 2004) and visualized with Circos v0.69.8 (Krzywinski et al., 2009) implemented via Mummer2Circos (https://github.com/metagenlab/mummer2circos).

### Morphological and physiological characterization

The cellular morphology of strain CJ426^T^ was observed using transmission electron microscopy. Cells were grown for two days at 30℃ on R2A agar. Growth was assessed on various agar media, including R2A agar, tryptic soy agar (TSA), Luria-Bertani (LB) agar, nutrient agar (NA), and marine agar (MA). To evaluate growth temperatures, cells were cultured on R2A agar at 4, 10, 15, 20, 25, 30, and 37℃. The pH growth range was determined using 0.1 M citrate buffer (pH 4.0–5.0), 0.1 M phosphate buffer (pH 6.0–8.0), and 0.1 M bicarbonate–carbonate buffer (pH 9.0–10.0), 0.05 M disodium hydrogen phosphate-sodium hydroxide buffer (pH 11.0–12.0), and 0.2 M potassium phosphate-sodium hydroxide buffer (pH 13.0) in R2A broth (Bates and Bower, 1956). Salt tolerance was tested by supplementing R2A broth with 0–3% (w/v) NaCl (at 1% intervals). Both the NaCl tolerance and pH range experiments were conducted at 30℃ for up to 3 days. Gram-staining was performed using a Gram-staining kit according to the manufacturer’s protocols (Sigma-Aldrich). Gliding motility was examined on semisolid R2A media containing 0.4% agar. Anaerobic growth was determined after two weeks of incubation at 30℃ on R2A agar using the GasPak Anaerobe Pouch System (BD). The production of flexirubin-type pigments was tested using a 20% KOH test and interpreted by changes in colony color. Oxidase and catalase activities were determined using oxidase reagent (bioMerieux) and a 3% (v/v) aqueous H_2_O_2_ solution, respectively. Hydrolysis of starch, cellulose, casein, and DNA was tested using media containing 1.0% (w/v) soluble starch, 0.2% (w/v) carboxymethyl (CM) cellulose, 3% (w/v) skimmed milk, and DNase test agar (BD), respectively. Results of the hydrolysis tests were evaluated after 2 days of incubation. Carbon source utilization and enzymatic activities of strain CJ426^T^ were determined using the API 20NE according to the manufacturer’s instructions.

### Chemotaxonomic analyses

The polar lipids of strain CJ426^T^ were extracted from 100 mg of freeze-dried cells cultured in R2A agar for two days at 30℃ and analyzed by two-dimensional silica gel thin-layer chromatography (TLC) (Tindall, 1990a, 1990b). Total lipids and specific functional group lipids were detected using molybdophosphoric acid, molybdenum blue spray, ninhydrin reagent and α-naphthol. The respiratory quinone was extracted as described by Minnikin et al. (1984) and determined by high performance liquid chromatography (HPLC) analysis using a reverse phase column (Apollo C18, 250 × 4.6 mm, 5 μm) (Collins, 1985). The quinones were eluted by a mixture of methanol and 2-propanol (4:1, v/v) with a flow rate of 1 ml/min and detected by UV absorbance at 270 nm. For fatty acid analysis, strain CJ426^T^ and related type strains were cultivated in R2A broth at 30℃ and harvested at the exponential growth phase. Fatty acids were saponified, methylated, and extracted following the standard protocol of the Sherlock Microbial Identification System (MIDI) version 6.2 and analyzed by the standard protocol of gas chromatography (Agilent Technologies). The cellular fatty acids were identified using the RTSBA6 database of the Microbial Identification System (Sasser, 1990).

### Antimicrobial susceptibility test

The antibiotic susceptibility of strain CJ426^T^ was evaluated using the disk diffusion assay with antibiotic impregnated disks (Lioflchem, Italy). The following antibiotic were used: amoxicillin (10 μg), cephalexin (30 μg), chloramphenicol (10 μg), ciprofloxacin (5 μg), clindamycin (2 μg), colistin (10 μg), erythromycin (10 μg), fosfomycin (200 μg), gentamicin (10 μg), linezolid (10 μg), meropenem (10 μg), rifampicin (5 μg), streptomycin (10 μg), sulfamethoxazole (50 μg), tetracycline (30 μg), trimethoprim (5 μg), tylosin (30 μg), vancomycin (5 μg). The susceptibility results were recorded as susceptible (S), intermediate (I), or resistant (R) based on the breakpoints for *Enterobacterales* and *Staphylococcus* spp. according to the CLSI supplement M100 (CLSI, 2025).

### Statistical analyses and visualization

Differences in genome size and G + C contents between the genera *Niabella* and *Paraniabella* were assessed using the t-test in R. A flower plot was visualized in R using the ‘plotrix’ packages. Scatter plot and box plots were generated in R using the ‘ggplot2’ package. The overall genomic relatedness indices (OGRI) heatmaps were visualized in R using the ‘pheatmap’ package. Pairwise correlations between ANI, AAI, and POCP values were calculated and visualized in R with ‘psych’ package. Functional annotation results on phylogenetic trees were visualized in R using the ‘ggplot2’, ‘ggtree’, ‘aplot’, ‘ggstance’, ‘patchwork’, and ‘ggstar’ packages.

### Nucleotide sequence accession numbers

The EMBL/DDBJ/GenBank accession numbers for the 16S rRNA gene sequence and genome sequence of strain CJ426^T^ are KF740309 and JBLOSY000000000, respectively.

## Results and Discussion

### Phylogeny based on 16S rRNA gene and whole genome sequences

**Fig. 1.**
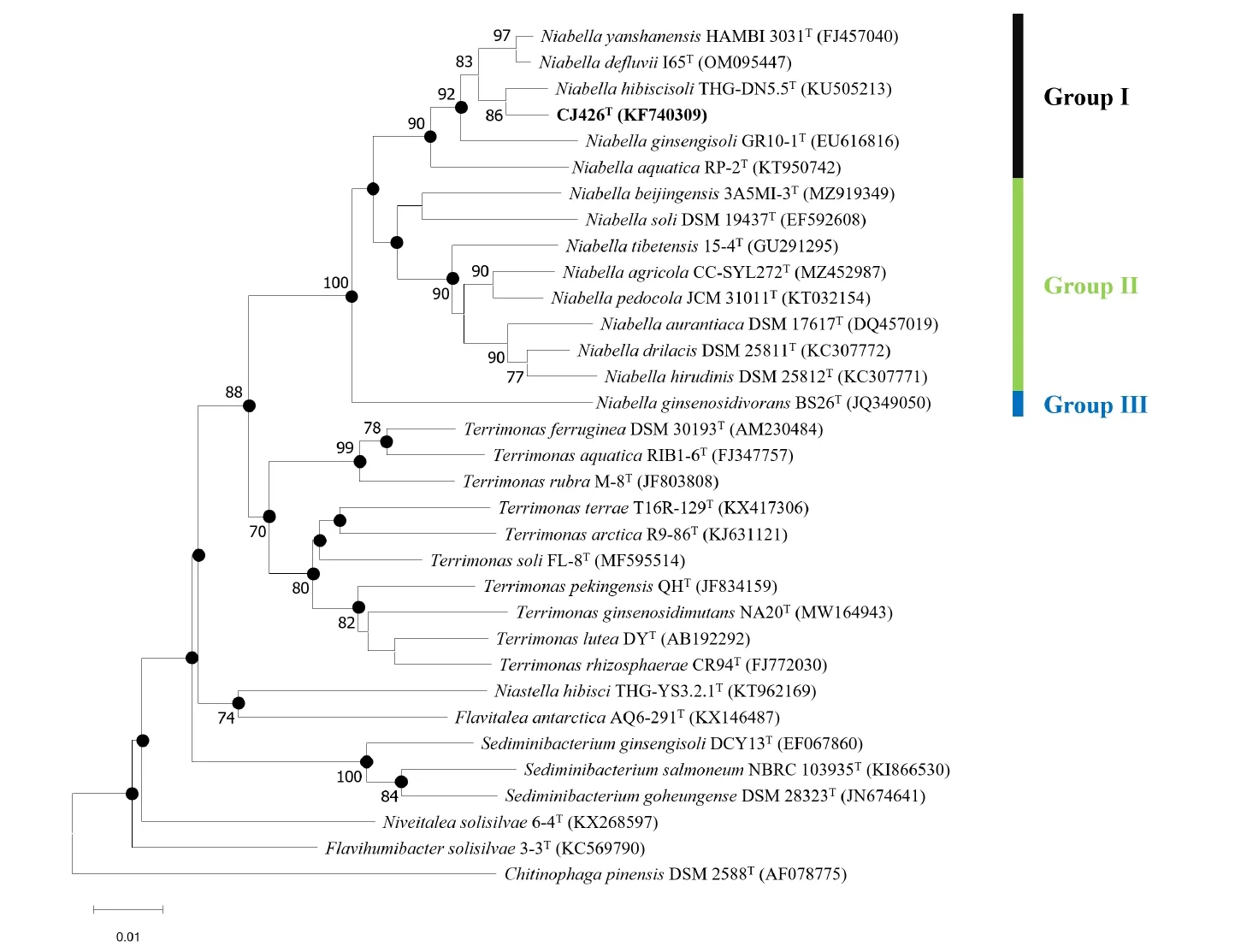
Neighbor-joining tree of strain CJ426^T^ and related type strains based on 16S rRNA gene sequences. Closed circles indicate nodes recovered in the three trees generated by neighbor-joining, maximum-likelihood and maximum-parsimony methods. Bootstrap values greater than 70% are shown at branch points based on 1,000 replicated datasets. *Chitinophaga pinensis* DSM 2588^T^ was used as an outgroup. Bar, 0.01 substitutions per nucleotide position.

The 16S rRNA gene sequence of strain CJ426^T^ showed the highest similarity (98.7%) to *N. hibiscisoli* THG-DN5.5^T^, followed by *N. yanshanensis* KACC 14980^T^ (98.1%), and *N. defluvii* I65^T^ (97.8%). In contrast, strain CJ426^T^ showed the lowest similarity (92.3%) to *N. tibetensis* 15-4^T^, followed by *N. beijingensis* 3A5MI-3^T^ (93.2%), and *N. agricola* CC-SYL272^T^ (93.2%). The pairwise 16S rRNA gene sequence identities, which are below the established thresholds for species (≤ 98.7%) (Kim et al., 2014) and genus delineation (≤ 94.5%; 95% confidence interval [CI]: 94.55–95.05%) (Yarza et al., 2014), suggest that strain CJ426^T^ can be proposed as a novel species and genus within the family *Chitinophagaceae* (Tables S1 and S2). Furthermore, based on the 16S rRNA gene similarity and the phylogenetic trees, the genus *Niabella* was divided into three preliminary groups (Fig. 1). According to the phylogenetic trees (Figs. 1, S1, and S2), strain CJ426^T^ formed a robust cluster with *N. hibiscisoli* THG-DN5.5^T^ and constructed a large clade with *N. aquatica* RP-2^T^, *N. ginsengisoli* GR10-1^T^, *N. defluvii* I65^T^, and *N. yanshanensis* HAMBI 3031^T^ (named Group Ⅰ). The remaining *Niabella* species formed another distinct clade (named Group Ⅱ) separated from the above species (Group Ⅰ), while *N. ginsenosidivorans* BS26^T^ formed a single distinct lineage (named Group Ⅲ). To elucidate more accurate taxonomic relationships among these groups, comprehensive analyses were performed using whole genome sequence data.

**Fig. 2.**
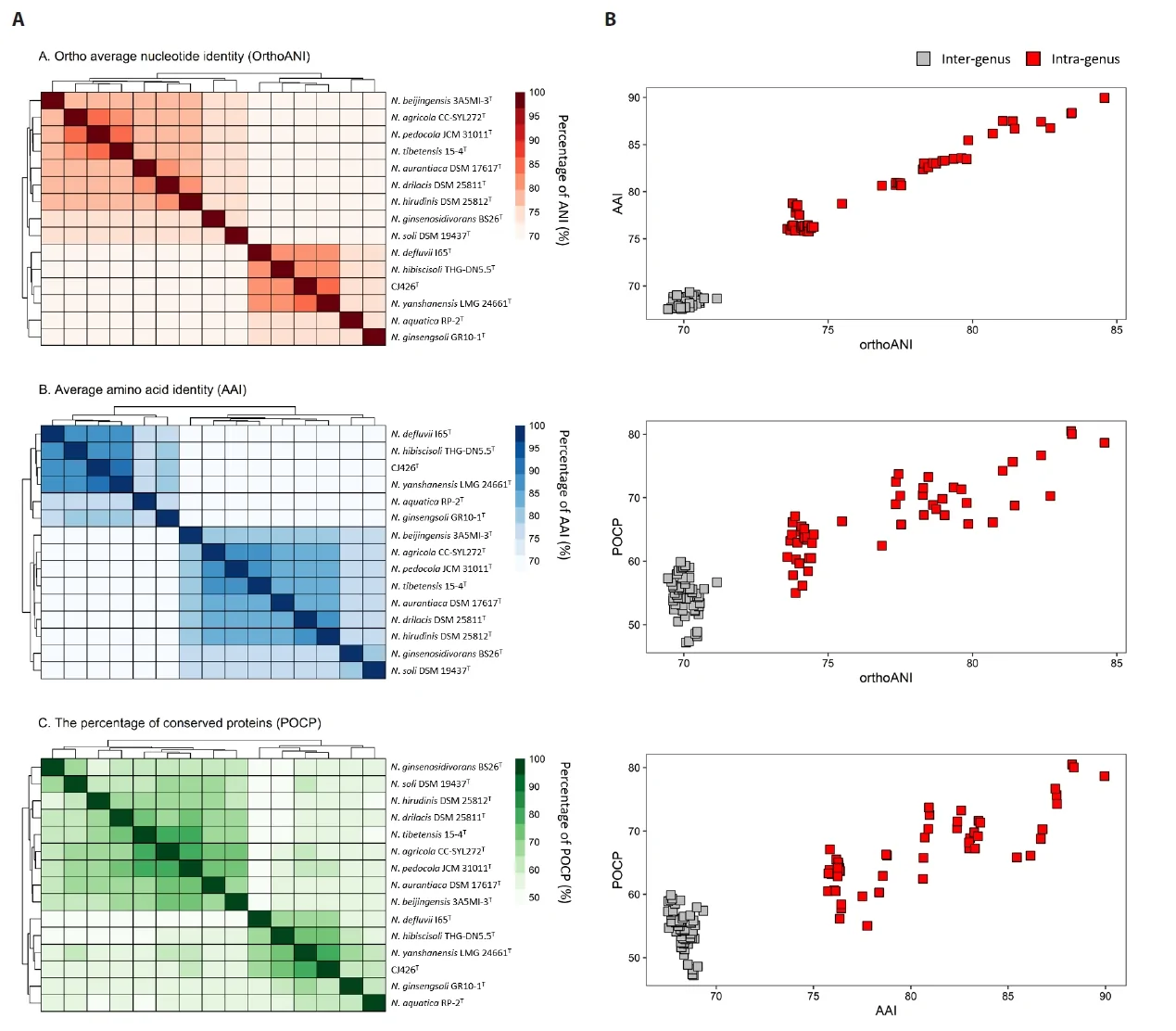
Comparative genomic features and overall genomic relatedness indices (OGRIs) of strain CJ426^T^ and related type strains. (A) The heatmaps displayed OrthoANI (red), AAI (blue), and POCP (green) values among genomes of the genera *Paraniabella* and *Niabella*. Dendrograms indicated clustering based on similarity metrics. Color gradients represented the percentage identity, with darker shades indicating higher similarity. (B) Pairwise correlation plots of OGRI values with proposed taxonomic thresholds for genus delineation.

**Fig. 3.**
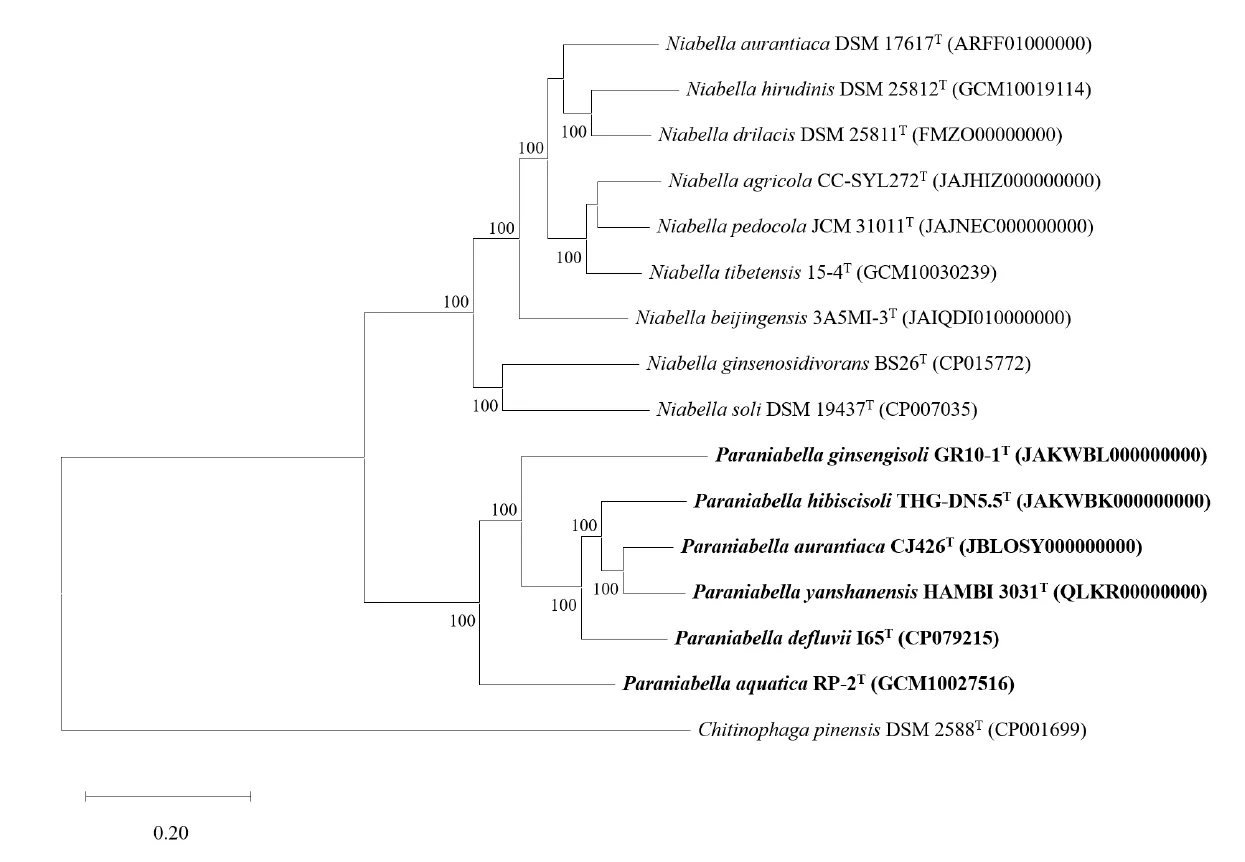
Phylogenomic tree of strain CJ426^T^ and related type strains based on 81 UBCGs. Bootstrap value greater than 70% are shown at branch points based on maximum-likelihood analysis of 1,000 replicated datasets. *Chitinophaga pinensis* DSM 2588^T^ was used as an outgroup. Bar, 0.01 substitutions per nucleotide position.

To analyze the overall genomic similarity between strain CJ426^T^ and its related type strains, ANI, AAI, dDDH, and POCP indices were calculated (Fig. 2A, Table S3). The ANI values between strain CJ426^T^ and all related type strains in the genus *Niabella* were 69.9% to 84.6% (Table S3). The dDDH values between strain CJ426^T^ and the related type strains were all below 30%. According to established thresholds, the ANI values below 95–96% and the dDDH values below 70% clearly suggest that strain CJ426^ᵀ^ represents a novel species within the family *Chitinophagaceae* at the genomic level (Chun et al., 2018; Meier-Kolthoff et al., 2013; Richter and Rosselló-Móra, 2009). For the genus delineation, the intra-group and inter-group ANI values were evaluated (Table 1). The intra-group ANI values within Group Ⅰ and Group Ⅱ were 73.6–84.6% and 73.7% to 83.4%, respectively. In contrast, the inter-group ANI values were 69.5–70.7% between Group Ⅰ and Group Ⅱ, 70.0–71.2% between Group Ⅰ and Group Ⅲ, and 74.2–75.5% between Group Ⅱ and Group Ⅲ. The ANI value range between Group Ⅰ and the other groups (Group Ⅱ and Group Ⅲ) was 69.5–71.2%, which was not only lower than the intra-group ANI values but also below the recently proposed genus boundary (an average of 73.10%, 95% CI: 72.50% to 73.70%) (Barco et al., 2020), suggesting that Group Ⅰ can be separated from the other groups at the genomic level. Additionally, AAI and POCP values were also used for genus delineation (Tables S3 and S4). The intra-group AAI values within Group Ⅰ and Group Ⅱ were 76.1–90.0% and 75.8–88.4%, respectively. In contrast, the inter-group AAI values were 67.5–69.3% between Group Ⅰ and Group Ⅱ, 67.9–68.9% between Group Ⅰ and Group Ⅲ, and 75.7–78.7% between Group Ⅱ and Group Ⅲ. These ranges were not only lower than those of the intra-group AAI values but also falls within the proposed threshold for genus boundary (an average of 70%) (Luo et al., 2014). The intra-group POCP values within Group Ⅰ and Group Ⅱ were 55.0–78.7% and 62.4–80.5%, respectively. In contrast, the inter-group AAI values were 47.2–59.9% between Group Ⅰ and Group Ⅱ, 48.2–56.7% between Group Ⅰ and Group Ⅲ, and 58.4–66.3% between Group Ⅱ and Group Ⅲ. Consequently, the POCP values between the members of Group Ⅰ and those of the other groups (Group Ⅱ and Group Ⅲ) had a lower range (47.2–59.9%) than the intra-group POCP values. Recent studies reported that the genera *Cnuella* and *Paracnuella* within the family *Chitinophagaceae* were separated with an inter-genus POCP value of 69.0% (Yuan et al., 2023). Meanwhile, pairwise comparisons of ANI, AAI, and POCP values indicated that the pair of ANI and AAI had a higher correlation coefficient (Spearman’s *ρ* = 0.88, *p* < 0.001) than other pairs (Fig. S3). Consequently, the results from ANI, AAI, and POCP analyses support that the members of Group Ⅰ are separable from the other groups (Fig. 2B). The relatively higher inter-group ANI, AAI, and POCP values between Group Ⅱ and Group Ⅲ, compared to those between Group I and the other groups, also suggest that Group Ⅱ and Group Ⅲ should be considered as a single group representing the genus *Niabella*. Based on genome-based analyses, the genus *Niabella* is highly divergent, highlighting the need for reclassification of the genus *Niabella*. Furthermore, the two phylogenomic trees showed that members of Group Ⅰ formed a distinct phylogenetic lineage separated from the other members of the genus *Niabella* (Figs. 3 and S4). Therefore, we propose that strain CJ426^T^ and closely related type strains within Group Ⅰ should be classified as the novel genus *Paraniabella*.

### Genomic features

**Fig. 4.**
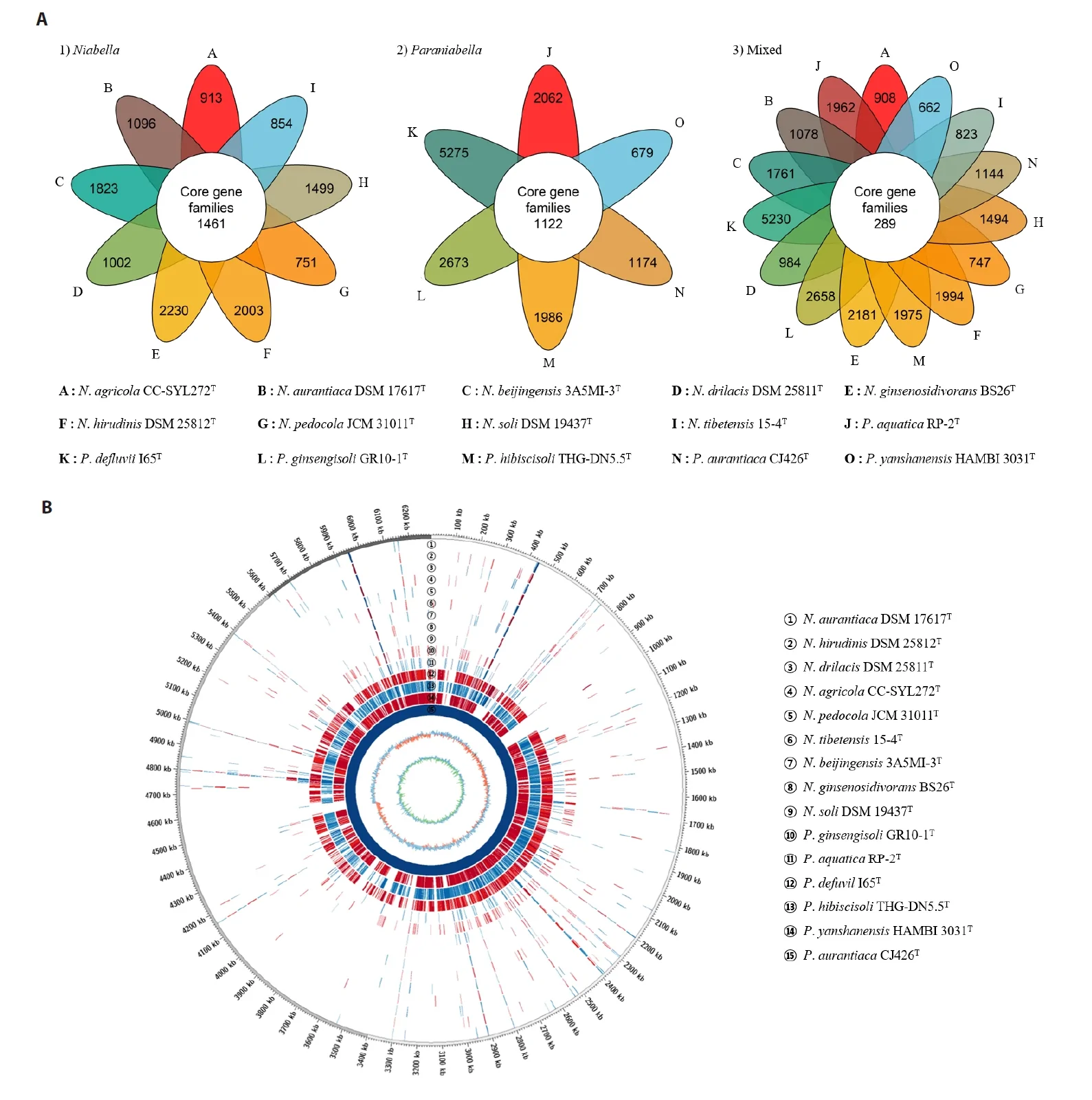
Comparative genomic analysis of strain CJ426^T^ and related type strains. (A) Pangenome analysis illustrates flower plots. The analysis shows the distribution of orthologous gene families calculated separately for 1) members of the genus *Niabella*, 2) members of the proposed genus *Paraniabella*, and 3) all strains from both genera combined. The numbers represent the counts of shared or unique orthologous gene families between the different strains. (B) Circular genome comparison of strain CJ426^T^ and all related type strains based on whole-genome alignment. The reference genome is shown in the innermost circle, with concentric rings representing the genomic features of each strain. Red and blue colors indicated regions of similarity.

The draft genome of strain CJ426^ᵀ^ comprised three contigs with no plasmids detected and had a total size of 6.3 Mbp (Fig. S5). The completeness of the genome assembly was evaluated to be 97.5% using BUSCO and 99.5% with a contamination level of 0.5% using CheckM. The genomic DNA G + C content of strain CJ426^T^ was calculated to be 41.9% based on genome sequence. The genome sequence of strain CJ426^T^ contained 5,310 CDSs, 45 tRNA genes, and 6 rRNA genes, including two identical copies of the 16S rRNA gene. A summary of the general genomic features of strain CJ426^ᵀ^ and related type strains is provided in Table 2. Comparison of general genomic features between the two genera revealed a significant difference in G + C contents (*p* < 0.001) but no significant difference in genome size (Fig. S6). The differences in G + C content values between species of the two genera *Niabella* (45.2–48.6%, an average of 46.8%) and *Paraniabella* (38.5–43.0%, an average of 41.7%) exceeded the cut-off of ≥ 1% within genus, also suggesting that strain CJ426^T^ and its related type strains should be classified as a novel genus, distinct from other *Niabella* type strains (Meier-Kolthoff et al., 2014). Pangenome analysis revealed that the genus *Niabella* and the proposed genus *Paraniabella* possessed 1,461 and 1,122 core orthologous genes, respectively (Fig. 4A). However, when all members of both genera were analyzed together, the number of core orthologous genes sharply decreased to 289 (Fig. 4A), further justifying the separation of these two genera. The whole genome sequence alignment analysis showed that strain CJ426^T^ and its related type strains within the genus *Paraniabella* exhibited higher similarities compared to other type strains within the genus *Niabella* (Fig. 4B).

According to functional annotation based on KEGG pathway, strain CJ426^T^ possessed 64 complete metabolic pathway modules (Fig. S7A), including well-conserved central carbohydrate metabolism pathways such as glycolysis, the Entner-Doudoroff pathway, pyruvate oxidation, gluconeogenesis, the citrate cycle, phosphoribosyl pyrophosphate biosynthesis and the pentose phosphate pathway (Table S4). Moreover, biosynthetic module for tryptophan was identified and consistent with phenotype production of indole. A single aminoglycoside O-nucleotidyltransferase (*aadS;ant(6)*), rifampin ADP-ribosyltransferase (*arr*), and two β-lactamase genes (subclass A2 and B3), which confer resistance to aminoglycosides, rifampin, and certain β-lactam antibiotics, were predicted in the genome sequence of strain CJ426^T^ (Fig. S7B). Strain CJ426^ᵀ^ encoded the highest number of antibiotic resistance genes compared to other related type strains: subclass A2 β-lactamase gene is exclusively present in its genome, which is known to hydrolyze penicillins and older cephalosporins (CfxA, CIA-1, CME-1, PER-1, and VEB-1) (Philippon et al., 2016). These results were consistent with phenotypic resistance to amoxicillin, meropenem, rifampicin and streptomycin (Table S5). Annotation of carbohydrate-active and proteolytic enzymes revealed that the genome of strain CJ426^ᵀ^ contained 239 carbohydrate-active enzymes, which were classified into two distinct families of CAZymes: 220 glycoside hydrolases and 19 polysaccharide lyases (Fig. S7B and Table S6). Additionally, the genome of strain CJ426^T^ encoded 62 proteolytic enzymes, including one aspartic peptidase, seven cysteine peptidases, one glutamic peptidase, 23 metallo peptidases, 29 serine peptidases, and one threonine peptidase (Fig. S7B and Table S7). Furthermore, the genome of strain CJ426^T^ contained several biosynthetic gene clusters (BGCs), including one lasso peptide BGC (8% similarity to known gene clusters), two copies of terpene BGCs (one showing 28% similarity to a known carotenoid BGC), two copies of flexirubin BGC (44% and 28% similarity to known gene clusters, respectively), one non-ribosomal peptide synthase (NRPS) BGC, and one type Ⅲ polyketide synthase (PKS) BGC. A summary of the BGCs of strain CJ426^ᵀ^ is provided in Table S8.

### Morphological, physiological, and biochemical characteristics

Cellular morphology was shown to be rod-shaped and non-flagellated (Fig. S7). Optimal growth media was R2A medium, but growth also occurred on TSA, NA, LB, and MA medium. Growth of strain CJ426^T^ occurred at 4–37℃ (optimum 30℃), at pH 5.0–12.0 (optimum pH 7.0), and at 0–2% concentration of NaCl (optimum 0%). Strain CJ426^T^ produced flexirubin-like pigments. The differential biochemical and physiological characteristics of strain CJ426^T^ and the type strains of related genera within the family *Chitinophagaceae* are summarized in Table 3. Additionally, differential characteristics among the members of the genera *Niabella* and *Paraniabella* are provided in Table S9. The genera *Niabella* and *Paraniabella* exhibit highly similar morphological, physiological and biochemical characteristics. Although phenotypic traits were not clearly distinguished between the two genera, all members of the proposed genus *Paraniabella* shared a consistent set of characteristics that differ from those of *Niabella*. For instance, all members of *Paraniabella* are positive for catalase activity and can assimilate D-glucose, L-arabinose, D-mannose, and D-maltose, but are unable to assimilate D-mannitol. In contrast, these traits are variable within the genus *Niabella*.

### Chemotaxonomic characteristics

The polar lipids of strain CJ426^T^ were identified as phosphatidylethanolamine, six unidentified aminolipids, and two unidentified lipids (Fig. S9). The respiratory quinone of strain CJ426^T^ was identified exclusively as menaquinone 7 (MK-7). The major fatty acids of strain CJ426^T^ included iso-C_15:0_ (38.2%), iso-C_15:0_ G (20.2%), and iso-C_17:0_ 3-OH (14.0%). As shown in Table 4, the fatty acid profile of strain CJ426^T^ was similar to those of closely related type strains within the family *Chitinophagaceae*, sharing major fatty acid components. These chemotaxonomic characteristics are consistent with those of other recognized species in the family *Chitinophagaceae*. The fatty acid profiles of strain CJ426^T^ and closely related type strains of the genera *Niabella* and *Paraniabella* are summarized in Table S10. Furthermore, a comparison of chemotaxonomic characteristics revealed that the genera *Niabella* and *Paraniabella* have similar fatty acid profiles, with the main difference being that the percentage of summed feature 3 tends to be higher in members of *Niabella* than in those of *Paraniabella*.

### Taxonomic conclusion

Phylogenetic analyses indicated that strain CJ426^T^ and its closely related type strains, *N. aquatica*, *N. defluvii*, *N. ginsengisoli*, *N. hibiscisoli*, and *N. yanshanensis*, formed distinct clades within the genus *Niabella* in all reconstructed trees, including NJ, ML, and MP phylogenetic trees based on the 16S rRNA gene sequences, as well as phylogenomic trees based on UBCGs and 290 SCGs concatenated alignment sequences, repectively. Moreover, the low OGRI values (ANI, AAI, POCP, and dDDH) bewteen the proposed *Paraniabella* group (including strain CJ426^T^) and the remaining members of the genus *Niabella* strongly support the reclassification of this group as a representative of a novel genus. Significant differences in G + C contents and core orthologous genes between these two groups also support the genus delineation. Based on polyphsic taxonomy study, strain CJ426^T^ is distinguished from closely related type strains on the basis of genome, chemical, molecular, and physiological characteristics, including metabolic pathways, antibiotic resistance genes, carbon source utilization and growth ranges. Futhermore, chemotaxonomic and phenotypic characteristics, such as respiratory quinone, polar lipid, fatty acid, and carbon source utilization, were concordant with the traits listed in the family *Chitinophagaceae* description.

Therefore, strain CJ426^T^ represents a novel species of a novel genus within the family *Chitinophagaceae*, for which the name *Paraniabella aurantiaca* gen. nov., sp. nov. is proposed. In addition, we propose that its phylogenetic neighbours *N. aquatica*, *N. defluvii*, *N. ginsengisoli*, *N. hibiscisoli*, and *N. yanshanensis* be reclassified into this novel genus as *Paraniabella aquatica* comb. nov., *Paraniabella defluvii* comb. nov., *Paraniabella ginsengisoli* comb. nov., *Paraniabella hibiscisoli* comb. nov., and *Paraniabella yanshanensis* comb. nov., respectively. On the basis of the genomic data obtained in this study, the description of the genus *Niabella* is emended.

### Description of *Paraniabella* gen. nov.

*Paraniabella* (Gr. prep. para, beside, near, like; N.L. fem. n. *Niabella*, a bacterial genus; N.L. fem. n. *Paraniabella*, a genus like *Niabella*).

Cells are Gram-negative, aerobic, non-motile, rod-shaped. Catalase activity is positive. D-Glucose, L-arabinose, D-mannose, and D-maltose assimilated, but D-mannitol is not. The major fatty acid (> 10%) are iso-C_15:0_, iso-C_15:1_ G, iso-C_17:0_ 3-OH, and the isoprenoid quinone is MK-7. A common major polar lipid is phosphatidylethanolamine. The genomic DNA G + C contents are 38.5–43.0%. The type species is *Paraniabella ginsengsoli*. The genus belongs to the family *Chitinophagaceae*.

### Description of *Paraniabella aurantiaca* sp. nov.

*Paraniabella aurantiaca* (au.ran.ti'a.ca. N.L. fem. adj. *aurantiaca*, orange-coloured).

Cells are Gram-negative, aerobic, non-motile, rod-shaped (1.5 µm long and 0.6 µm wide. Colonies on R2A agar are circular, opaque, orange-coloured. Growth occurs at 4–37°C (optimum 30°C), at pH 5.0–12.0 (optimum pH 7.0) and with 0–2% NaCl (optimum 0%). Flexirubin-type pigments are produced. Did not show any motility on 0.4% agar. Casein, cellulose, gelatin, esculin ferric citrate and starch are hydrolyzed, but DNA is not. Positive for catalase but negative for oxidase. Nitrate and nitrite are not reduced. Indole production is detected. D-Glucose is not fermented. Enzyme activities for arginine dihydrolase and β-galactosidase are positive, but urease is not. According to the API 20NE test, D-glucose, L-arabinose, D-mannose, *N*-acetyl-glucosamine, and D-maltose are assimilated, but D-mannitol, potassium gluconate, capric acid, adipic acid, malic acid, trisodium citrate and phenylacetic acid are not. The predominant polar lipid is phosphatidylethanolamine. The major fatty acid is iso-C_15:0_, iso-C_15:1_ G, and iso-C_17:0_ 3-OH. The isoprenoid quinone is MK-7. The type strain is CJ426^T^ (= KACC 23908^T^ = JCM 37728^T^), isolated from ginseng soil in Korea. The genomic DNA G + C content of the type strain is 41.9%. The EMBL/DDBJ/GenBank accession numbers for the 16S rRNA gene sequence and the whole genome sequence of strain CJ426^T^ are KF740309 and JBLOSY000000000, respectively.

### Description of *Paraniabella aquatica* comb. nov.

*Paraniabella aquatica* (a.qua′ti.ca. L. fem. adj. aquatica aquatic, from water).

Basonym: *Niabella aquatica* Siddiqi and Im. 2016

The description is identical to that given for *N. aquatica* (Siddiqi and Im, 2016). Phylogenomic analysis and overall genome relatedness indices strongly support the placement of this species within the novel genus *Paraniabella*. The type strain is RP-2^T^ (= KACC 18623^T^ = JCM 30952^T^).

### Description of *Paraniabella defluvii* comb. nov.

*Paraniabella defluvii* (de. flu′vi.i. L. gen. n. defluvii, of sewage).

Basonym: *Niabella defluvii* Min et al. 2024

The description is identical to that given for *N. defluvii* (Min et al., 2024). Phylogenomic analysis and overall genome relatedness indices strongly support the placement of this species within the novel genus *Paraniabella*. The type strain is I65^T^ (= KACC 22647^T^ = JCM 35315^T^).

### Description of *Paraniabella ginsengisoli* comb. nov.

*Paraniabella ginsengisoli* (gin.seng.i.so′li. N.L. n. ginsengum ginseng; L. n. solum soil; N.L. gen. n. ginsengisoli of soil from a ginseng field, the source of the type strain).

Basonym: *Niabella ginsengisoli* Weon et al. 2009

The description is identical to that given for *N. ginsengisoli* (Weon et al., 2009). Phylogenomic analysis and overall genome relatedness indices strongly support the placement of this species within the novel genus *Paraniabella*. The type strain is GR10-1^T^ (= KACC 13021^T^ = JCM 15444^T^).

### Description of *Paraniabella hibiscisoli* comb. nov.

*Paraniabella hibiscicoli* (hi.bis.ci.so′li. N.L. n. hibiscus Rose of Sharon/ Hibiscus syriacus; L. n. solum soil; N.L. gen. n. hibis cisoli of soil of a Rose of Sharon garden, the source of the type strain).

Basonym: *Niabella hibiscisoli* Ngo et al. 2017

The description is identical to that given for *N. hibiscisoli* (Ngo et al., 2017). Phylogenomic analysis and overall genome relatedness indices strongly support the placement of this species within the novel genus *Paraniabella*. The type strain is THG-DN5.5^T^ (= KACC 18857^T^ = CCTCC AB 2016086^T^).

### Description of *Paraniabella yanshanensis* comb. nov.

*Paraniabella yanshanensis* (yan.sha.nen′sis. N.L. fem. adj. yanshanensis of Yanshan, an emblem for Hebei Province, where the type strain was isolated).

Basonym: *Niabella yanshanensis* Wang et al. 2009

The description is identical to that given for *N. yanshanensis* (Wang et al., 2009). Phylogenomic analysis and overall genome relatedness indices strongly support the placement of this species within the novel genus *Paraniabella*. The type strain is CCBAU 05354^T^ (= LMG 24661^T^ = HAMBI 3031^T^).

### Emended description of the genus *Niabella*Dai et al. 2011

The description of the genus is as given by Dai et al. (2011), with the following modifications. Based on the genome sequences, the DNA G + C content of the members of the genus *Niabella* ranges from 45.2 to 48.6%.

## Figures and Tables

**Table 1.** Comparison of intra- and inter-group OGRI values

OGRI values (%)		Group Ⅰ	Group Ⅱ	Group Ⅲ
Group Ⅰ	ANI	73.6–84.6		
	AAI	76.1–90.0		
	POCP	55.0–78.7		
Group Ⅱ	ANI	69.5–70.7	73.7–83.4	
	AAI	67.5–69.3	75.8–88.4	
	POCP	47.2–59.9	62.4–80.5	
Group Ⅲ	ANI	70.0–71.2	74.2–75.5	NA
	AAI	67.9–68.9	75.7–78.7	
	POCP	48.2–56.7	58.4–66.3	

NA, not applicable

**Table 2.** General genomic features of strain CJ426^T^ and related type strains within the genera *Niabella* and *Paraniabella*

No	Type strains	Accession number	Size (Mbp)	GC contents (%)	Number of			
					contigs	CDSs	rRNA	tRNA
1	*N. agricola* CC-SYL272^T^	JAJHIZ000000000^†^	6.3	47.1	3	5,286	6	48
2	*N. aurantiaca* DSM 17617^T^	ARFF01000000^†^	5.9	48.6	25	4,870	12	50
3	*N. beijingensis* 3A5MI-3^T^	JAIQDI010000000^†^	6.6	47.1	3	5,604	3	43
4	*N. drilacis* DSM 25811^T^	FMZO00000000^†^	6.1	47.8	45	5,189	3	46
5	*N. ginsenosidivorans* BS26^T^	CP015772^†^	5.6	44.5	1	4,741	6	44
6	*N. hirudinis* DSM 25812^T^	GCM10019114^‡^	5.4	46.9	1	6,674	6	53
7	*N. pedocola* JCM 31011^T^	JAJNEC000000000^†^	6.5	47.3	11	5,310	3	42
8	*N. soli* DSM 19437^T^	CP007035^†^	4.7	45.2	1	3,888	6	41
9	*N. tibetebsis* 15-4^T^	GCM10030239^‡^	6.6	47.0	54	5,412	6	48
10	*P. aquatica* RP-2^T^	GCM10027516^‡^	5.4	42.0	40	4,539	3	38
11	*P. aurantiaca* CJ426^T^	JBLOSY000000000^*^	6.3	41.9	3	5,310	6	45
12	*P. defluvii* I65^T^	CP079215^†^	6.1	43.0	1	9,449	6	43
13	*P. ginsengisoli* GR10-1^T^	JAKWBL000000000^†^	4.5	38.5	5	5,443	6	44
14	*P. hibiscisoli* THG-DN5.5^T^	JAKWBK000000000^†^	6.1	42.1	5	8,185	6	42
15	*P. yanshanensis* HAMBI 3031^T^	QLKR00000000^†^	5.5	42.7	25	4,607	3	41

All data analyses were performed in this study.

* Genome sequencing performed in this study.

† Genome sequences were obtained from the National Institute of Technology and Evaluation.

‡ Genome sequences were obtained from the Type Strains Genome Database.

**Table 3.** Differential characteristics of strain CJ426^T^ and related type strains of the family *Chitinophagaceae*. Strains: 1, CJ426^T^ (this study); 2, *N. aurantiaca* DSM 17617^T^ (Kim et al., 2007); 3, *Terrimonas ferruginea* DY^T^ (Jin et al., 2013; Xie and Yokota, 2006); 4, *Niastella hibisci* THG-YS3.2.1^T^ (Yan et al., 2016); 5, *Sediminibacterium salmoneum* NBRC 103935^T^ (Kim et al., 2013; Qu and Yuan, 2008); 6, *Niveitalea solisilvae* 6-4^T^ (Hyeon et al., 2017); 7, *Flavihumibacter solisilvae* 3-3^T^ (Lee et al., 2014). Symbols: +, Positive; W, weakly positive; -, negative; ND, data not available. All strains are positive for activity of β-galactosidase, and esculin hydrolysis. All strains are negative for the assimilation of D-mannitol, potassium gluconate, capric acid, adipic acid, malic acid, trisodium citrate and phenylacetic acid.

Characteristic	1	2	3	4	5	6	7
Isolation source^†^	Soil	Soil	Soil	Soil	Sediment	Soil	Soil
Colony color^‡^	O	O	SR	Y	SP	W	Y
Cell morphology	Rod	Rod	Rod	Rod	Rod	Rod	Rod
Cell motility	-	-	-	-	+	-	-
Growth
Temperature (°C)	4–37	10–35	10–37	10–40	18–37	20–35	20–37
pH	5–12	5–8	ND	6–8	6.0–7.5	6.0–9.0	5.5–9.5
NaCl (%, w/v)	0–2	0–3	0–1	0–1	0–1	0	0–0.5
Nirate reduction	-	-	+	-	-	+	-
Indole production	+	+	-	-	-	-	-
Fermentation of							
Glucose	-	-	-	-	+	-	-
Hydrolysis of							
Casein	+	+	-	-	-	+	+
DNA	-	-	+	-	-	ND	ND
Starch	+	-	+	-	-	-	-
CM-Cellulose	+	+	-	+	-	ND	ND
Gelatin (API 20NE)	+	-	+	+	-	-	+
Enzyme of activity of:							
Catalase	+	+	w	+	+	-	+
Oxidase	-	-	+	-	+	+	+
Urease	+	-	-	-	-	-	-
Arginine dihydrolase	+	-	-	-	-	-	-
Assimilation of:							
D-Glucose	+	+	+	+	+	-	+
L-Arabinose	+	+	+	-	-	-	+
D-Mannose	+	+	w	-	+	-	-
*N*-Acetyl-glucosamine	+	+	+	-	-	-	+
D-Maltose	+	+	+	-	+	-	+
G + C content (mol%)	41.9^*^	45.0	48.9	45.3	40.6	45.8	49.5
Major polar lipids^⁑^	PE	ND	PE	PME, PE	PE	PE	PE

† 1, Ginseng; 2, Greenhouse; 3, Unkown; 4, Rhizosphere; 5, Eutrophic reservoir; 6, Forest; 7, Forest

‡ O,Orange; SR, Salmon red; Y, Yellow; BY, Bright yellow; SP, Salmon pink; W, White

* Data from genome sequence

⁑ PME, phosphatidylmethylethanolamine; PE, phosphatidylethanolamine

**Table 4.** Fatty acid compositions (%) of strain CJ426^T^ and related type strains of the family *Chitinophagaceae*. Strains: 1, CJ426^T^ (this study); 2, *N. aurantiaca* DSM 17617^T^ (Kim et al., 2007); 3, *Terrimonas ferruginea* DY^T^ (Xie and Yokota, 2006); 4, *Niastella hibisci* THG-YS3.2.1^T^ (Yan et al., 2016); 5, *Sediminibacterium salmoneum* NBRC 103935^T^ (Qu and Yuan, 2008); 6, *Niveitalea solisilvae* 6-4^T^ (Hyeon et al., 2017); 7, *Flavihumibacter solisilvae* 3-3^T^ (Lee et al., 2014). Data are percentages of the total fatty acids (components < 1.0% in all strains are not shown). TR, trace amounts (< 1.0%); -, not detected; Major components (> 10.0%) are highlighted in bold type.

Fatty acid	1	2	3	4	5	6	7
Straight								
C_14:0_	1.1	-	TR	TR	-	-	-
C_15:0_	-	-	2.7	-	TR	-	-
C_16:0_	5.9	3.5	1.7	8.3	1.2	2.6	1.7
C_18:0_	-	-	-	3.7	-	-	-
Branched								
anteiso-C_12:0_	-	-	-	2.3	-	-	-
iso-C_13:0_	-	-	TR	-	-	-	1.3
iso-C_14:0_	-	-	1.1	1.6	2.5	1.0	-
iso-C_15:0_	**38.2**	**33.7**	**28.4**	**14.3**	**17.5**	**34.0**	**39.8**
iso-C_15:1_	-	-	**26.2**	-	-	-	-
anteiso-C_15:0_	-	1.6	TR	5.0	9.5	**11.2**	2.5
iso-C_15:1_ G	**20.2**	**22.3**	-	9.3	**24.1**	**12.6**	**24.2**
anteiso-C_15:1_ A	-	-	-	1.6	7.4	2.0	-
iso-C_16:0_	-	-	1.4	3.5	1.1	2.4	-
iso-C_16:0_ G	-	-	-	2.2	-	-	-
Unsaturated								
C_14:1_ *ω*5*c*	-	-	-	-	-	1.0	-
C_16:1_ *ω*5*c*	1.4	-	-	-	TR	6.6	6.1
C_17:1_ *ω6c*	-	-	-	3.3	-	-	-
C_18:3_ *ω6c* (6,9,12)	-	-	-	2.5	-	-	-
Hydroxy								
C_15:0_ 2-OH	-	-	TR	1.1	1.9	-	-
iso-C_15:0_ 3-OH	2.8	2.9	2.2	-	7.4	2.8	3.7
C_16:0_ 3-OH	2.0	2.4	2.5	1.5	1.8	1.6	-
iso-C_16:0_ 3-OH	-	-	TR	6.1	7.7	1.0	-
C_17:0_ 2-OH	-	-	-	2.3	2.2	1.2	-
iso-C_17:0_ 3-OH	**14.0**	**15.5**	**15.3**	**19.1**	7.7	**12.2**	3.0
Summed feature 3^†^	7.2	**10.6**	**11.2**	**11.1**	TR	4.4	**11.1**

† Summed features represent groups of two or three fatty acids that could not be separated by GLC with the MIDI system. Summed feature 3 comprises C_16:1_ *ω7c* and/or C_16:1_ *ω6c*.

## Supplementary Information

Table S1. 16S rRNA gene similarity between strain CJ426^T^ and related type strains

Table S2. Comparison 16S rRNA gene similarity of the type strains within the genus *Niabella*

Table S3. OrthoANI, AAI, POCP and dDDH values between strain CJ426^T^ and related type strains

Table S4. List of metabolic pathway modules based on KEGG databases

Table S5. Antimicrobial susceptibility (inhibition zone diameter; mm) of *Paraniabella aurantiaca* CJ426^T^

Table S6. The number of predicted family domains of carbohydrate-active enzymes based on the CAZy database for CJ426^T^ and related type strains

Table S7. The number of predicted family domains of proteolytic enzymes based on the MEROPS database for CJ426^T^ and related type strains. -, not detected

Table S8. The information of biosynthetic gene clusters in strain CJ426^T^

Table S9. Characteristics of strain CJ426^T^ and the closely related type strains within the genera *Niabella* and *Paraniabella*. Strains: 1, CJ426^T^; 2, *P. hibiscisoli* THG-DN5.5^T^; 3, *P. yanshanensis* HAMBI 3031^T^; 4, *P. defluvii* I65^T^; 5, *P. ginsengisoli* GR10-1^T^; 6, *P. aquatica* RP-2^T^; 7, *N. beijingensis* 3A5MI-3^T^; 8, *N. soli* DSM 19437^T^; 9, *N. tibetensis* 15-4^T^; 10, *N. agricola* CC-SYL272^T^; 11, *N. pedocola* JCM 31011^T^; 12, *N. aurantiaca* DSM 17617^T^; 13, *N. drilacis* DSM 25811^T^; 14, *N. hirudinis* DSM 25812^T^; 15, *N. ginsenosidivorans* BS26^T^. The data for strain CJ426 was obtained from this study, while the data for other strains were taken from published literature. +, Positive; w, weakly positive; -, negative; ND, data not available

Table S10. Fatty acid composition of strain CJ426^T^ and related type strains. Strains: 1, CJ426^T^; 2, *P. hibiscisoli* THG-DN5.5^T^; 3, *P. yanshanensis* HAMBI 3031^T^; 4, *P. defluvii* I65^T^; 5, *P. ginsengisoli* GR10-1^T^; 6, *P. aquatica* RP-2^T^; 7, *N. beijingensis* 3A5MI-3^T^; 8, *N. soli* DSM 19437^T^; 9, *N. tibetensis* 15-4^T^; 10, *N. agricola* CC-SYL272^T^; 11, *N. pedocola* JCM 31011^T^; 12, *N. aurantiaca* DSM 17617^T^; 13, *N. drilacis* DSM 25811^T^; 14, *N. hirudinis* DSM 25812^T^; 15, *N. ginsenosidivorans* BS26^T^. -, Fatty acid amounts < 1.0% or not detected. The data for strain CJ426 was obtained from this study, while the data for other strains were taken from published literature.

Fig. S1. Maximum-likelihood phylogenetic tree of strain CJ426^T^ and related type strains based on 16S rRNA gene sequences. Bootstrap values greater than 70% are shown at branch points based on maximum-likelihood analysis of 1,000 replicated datasets. *Chitinophaga pinensis* DSM 2588^T^ was used as an outgroup. Bars, 0.050 substitutions per nucleotide position.

Fig. S2. Maximum-parsimony (MP) tree based on 16S rRNA gene sequences showing the phylogenetic relationships between strain CJ426^T^ and related type strains of the Genera. Bootstrap values greater than 70% are shown at branch points based on 1,000 replicated datasets. *Chitinophaga pinensis* DSM 2588^T^ was used as an outgroup.

Fig. S3. Pairwise correlations of OrthoANI, AAI, and POCP calculated from the genomes of *Niabella* and *Paraniabella* type strains. The pairwise correlation calculated using Spearman’s Rank Correlation Coefficient. ^***^*p* < 0.001.

Fig. S4. Phylogenomic tree of strain CJ426^T^ and related type strains based on the concatenated sequences of 290 core genes. Bootstrap values are shown at branch points based on analyses of 1,000 replicated datasets. *Chitinophaga pinensis* DSM 2588^T^ was used as an outgroup.

Fig. S5. The draft genome map of *Paraniabella aurantiaca* CJ426^T^. From inner to outer tracks: The first track shows the GC skew values with dark green indicating lower than zero and green indicating higher than zero. The second track represents the GC con tent. The third, fourth and fifth tracks display the locations of COG, CDS, tRNA and rRNA on reverse strand of the genome, respectively. The sixth, seventh, and eighth tracks show the locations of tRNA, rRNA, CDS, and COG on forward strand of the genome, respectively.

Fig. S6. Scatter and box plots for comparison of genome size (Mbp) and G + C content (%) between the genera *Paraniabella* and *Niabella*. Statistical significance was assessed using a t-test (NS, not significant; ^***^*p* < 0.001).

Fig. S7. Functional genomic analysis of strain CJ426ᵀ and related type strains. (A) Heatmap showed the presence or absence of metabolic pathway modules based on KEGG annotations. (B) Comparative analysis of functional genes among the type strains based on various databases. The heatmap showed the presence or absence of antibiotic resistance genes. The Bar plot represents the distribution of proteolytic enzymes and CAZYmes. The dendrogram was a phylogenomic tree based on 81 UBCG.

Fig. S8. Transmission electron micrograph (TEM) of strain CJ426^T^. Cells were grown on R2A agar for 2 days at 30℃.

Fig. S9. Two-dimensional thin-layer chromatography of the polar lipids of strain CJ426^T^. First dimension, chloroform/methanol/water (65:25:4); second dimension, chloroform/methanol/acetic acid/water (80:12:15:4). Abbreviations: PE, phosphatidylethanolamine; AL, amino lipid; L, unidentified lipid.
