# Supplementary material for: Genome-based classification of Paraniabella aurantiaca gen. nov., sp. nov., isolated from soil and taxonomic reclassification of five species within the genus Niabella
Source: J Microbiol. 2025 Oct 31;63(10):e2505005. doi: 10.71150/jm.2505005 (PMC13576996; doi:10.71150/jm.2505005)
Supplement: Supplementary file 1 [file jm-2505005-Supplementary-Fig-S1.pdf]

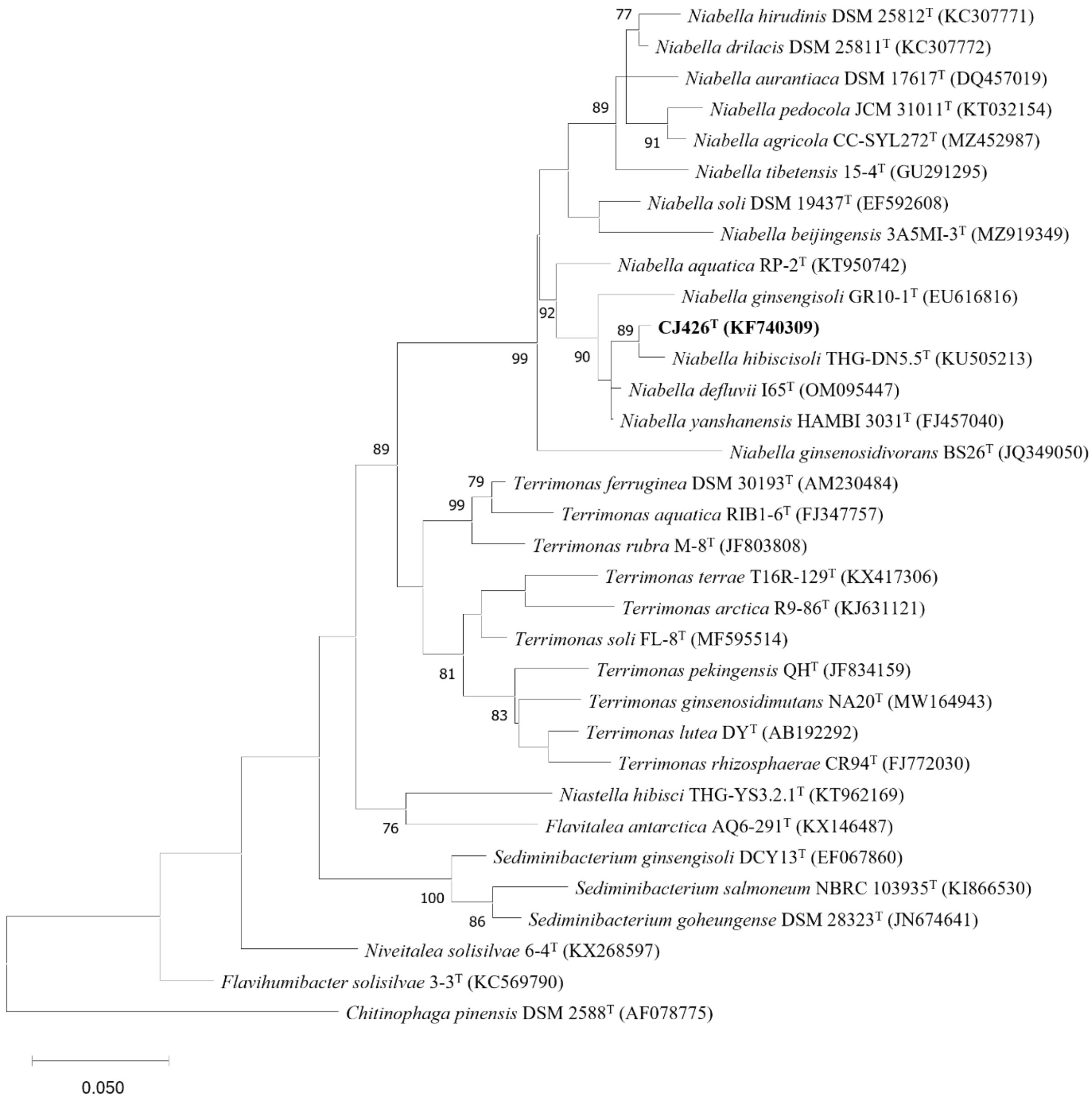

Fig. S1. Maximum-likelihood phylogenetic tree of strain CJ426^{T} and related type strains based on 16S rRNA gene sequences. Bootstrap values greater than 70% are shown at branch points based on maximum-likelihood analysis of 1,000 replicated datasets. *Chitinophaga pinensis* DSM 2588^{T} was used as an outgroup. Bars, 0.050 substitutions per nucleotide position.
