# Supplementary material for: Genome-based classification of Paraniabella aurantiaca gen. nov., sp. nov., isolated from soil and taxonomic reclassification of five species within the genus Niabella
Source: J Microbiol. 2025 Oct 31;63(10):e2505005. doi: 10.71150/jm.2505005 (PMC13576996; doi:10.71150/jm.2505005)
Supplement: Supplementary file 2 [file jm-2505005-Supplementary-Fig-S2.pdf]

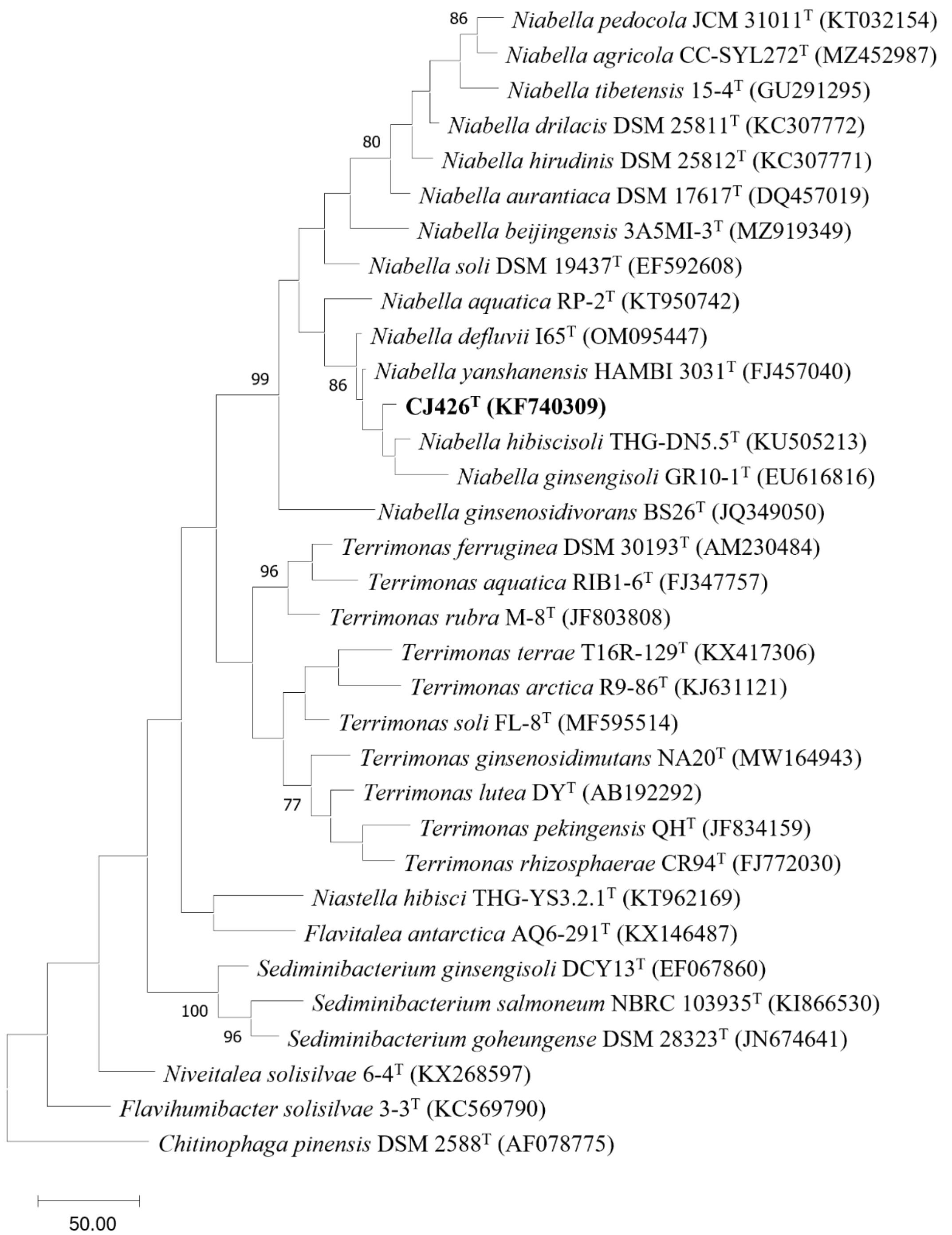

Fig. S2. Maximum-parsimony (MP) tree based on 16S rRNA gene sequences showing the phylogenetic relationships between strain CJ426^{T} and related type strains of the Genera. Bootstrap values greater than 70% are shown at branch points based on 1,000 replicated datasets. *Chitinophaga pinensis* DSM 2588^{T} was used as an outgroup.
