# Supplementary material for: Genome-based classification of Paraniabella aurantiaca gen. nov., sp. nov., isolated from soil and taxonomic reclassification of five species within the genus Niabella
Source: J Microbiol. 2025 Oct 31;63(10):e2505005. doi: 10.71150/jm.2505005 (PMC13576996; doi:10.71150/jm.2505005)
Supplement: Supplementary file 3 [file jm-2505005-Supplementary-Fig-S3.pdf]

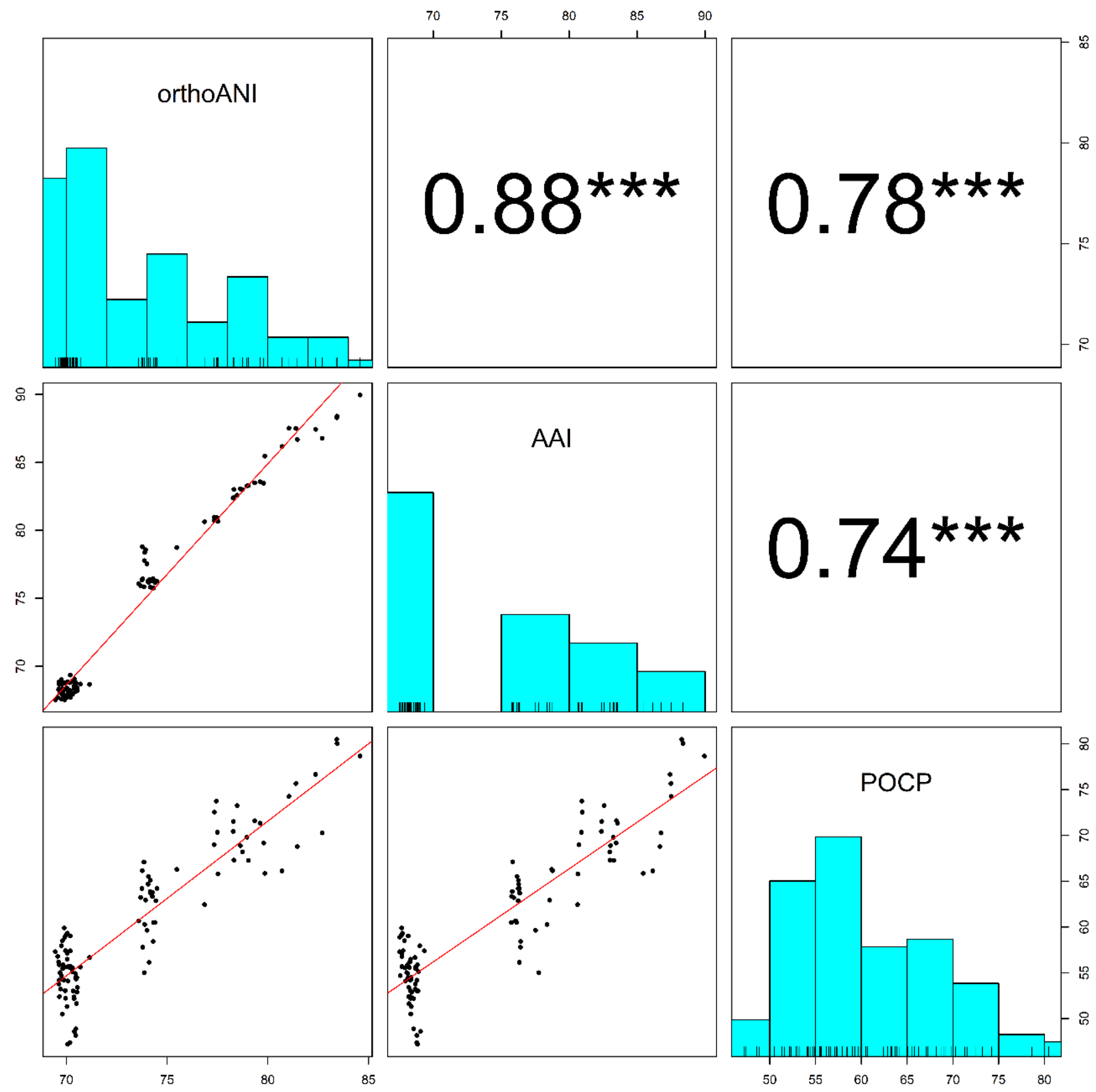


**Fig. S3.** Pairwise correlations of OrthoANI, AAI, and POCP calculated from the genomes of *Niabella* and *Paraniabella* type strains. The pairwise correlation calculated using Spearman's Rank Correlation Coefficient. ***$p < 0.001$.
