# Supplementary material for: Genome-based classification of Paraniabella aurantiaca gen. nov., sp. nov., isolated from soil and taxonomic reclassification of five species within the genus Niabella
Source: J Microbiol. 2025 Oct 31;63(10):e2505005. doi: 10.71150/jm.2505005 (PMC13576996; doi:10.71150/jm.2505005)
Supplement: Supplementary file 4 [file jm-2505005-Supplementary-Fig-S4.pdf]

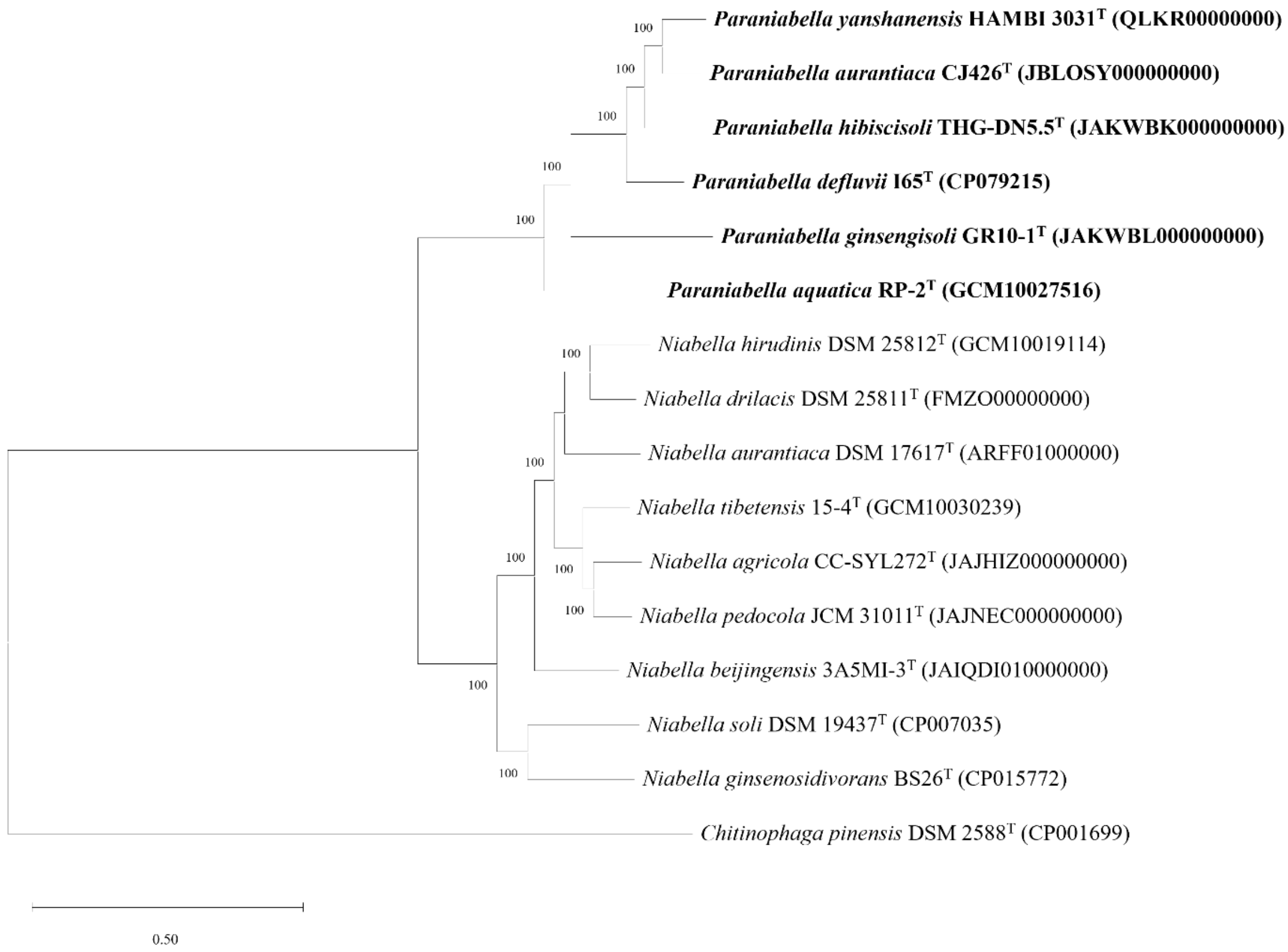

Fig. S4. Phylogenomic tree of strain CJ426^T and related type strains based on the concatenated sequences of 290 core genes. Bootstrap values are shown at branch points based on analyses of 1,000 replicated datasets. *Chitinophaga pinensis* DSM 2588^T was used as an outgroup.
