# Supplementary material for: Genome-based classification of Paraniabella aurantiaca gen. nov., sp. nov., isolated from soil and taxonomic reclassification of five species within the genus Niabella
Source: J Microbiol. 2025 Oct 31;63(10):e2505005. doi: 10.71150/jm.2505005 (PMC13576996; doi:10.71150/jm.2505005)
Supplement: Supplementary file 5 [file jm-2505005-Supplementary-Fig-S5.pdf]

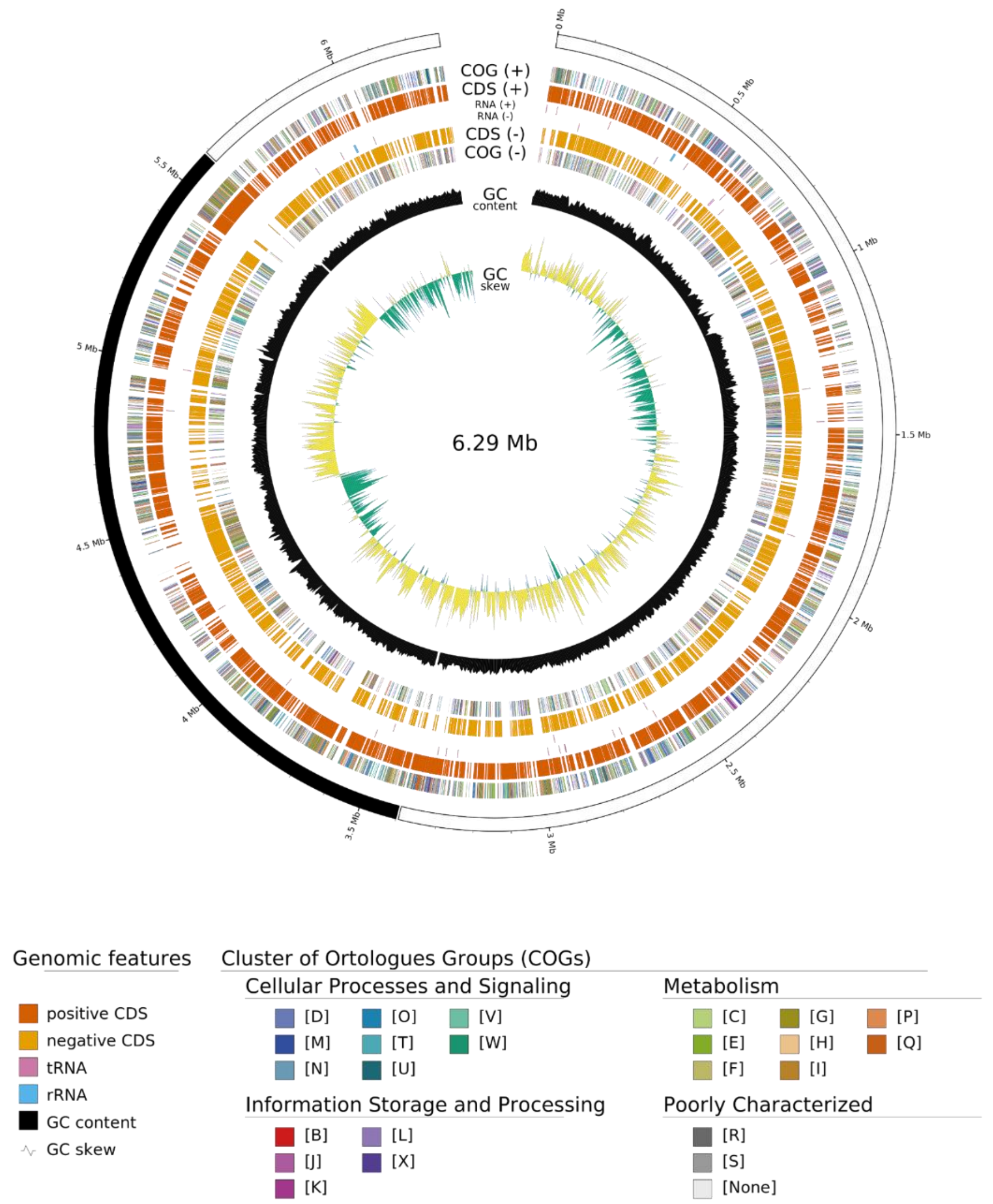

Fig. S5. The draft genome map of *Paraniabella aurantiaca* CJ426^{T}. From inner to outer tracks: The first track shows the GC skew values with dark green indicating lower than zero and green indicating higher than zero. The second track represents the GC con tent. The third, fourth and fifth tracks display the locations of COG, CDS, tRNA and rRNA on reverse strand of the genome, respectively. The sixth, seventh, and eighth tracks show the locations of tRNA, rRNA, CDS, and COG on forward strand of the genome, respectively.
