# Supplementary material for: Genome-based classification of Paraniabella aurantiaca gen. nov., sp. nov., isolated from soil and taxonomic reclassification of five species within the genus Niabella
Source: J Microbiol. 2025 Oct 31;63(10):e2505005. doi: 10.71150/jm.2505005 (PMC13576996; doi:10.71150/jm.2505005)
Supplement: Supplementary file 6 [file jm-2505005-Supplementary-Fig-S6.pdf]

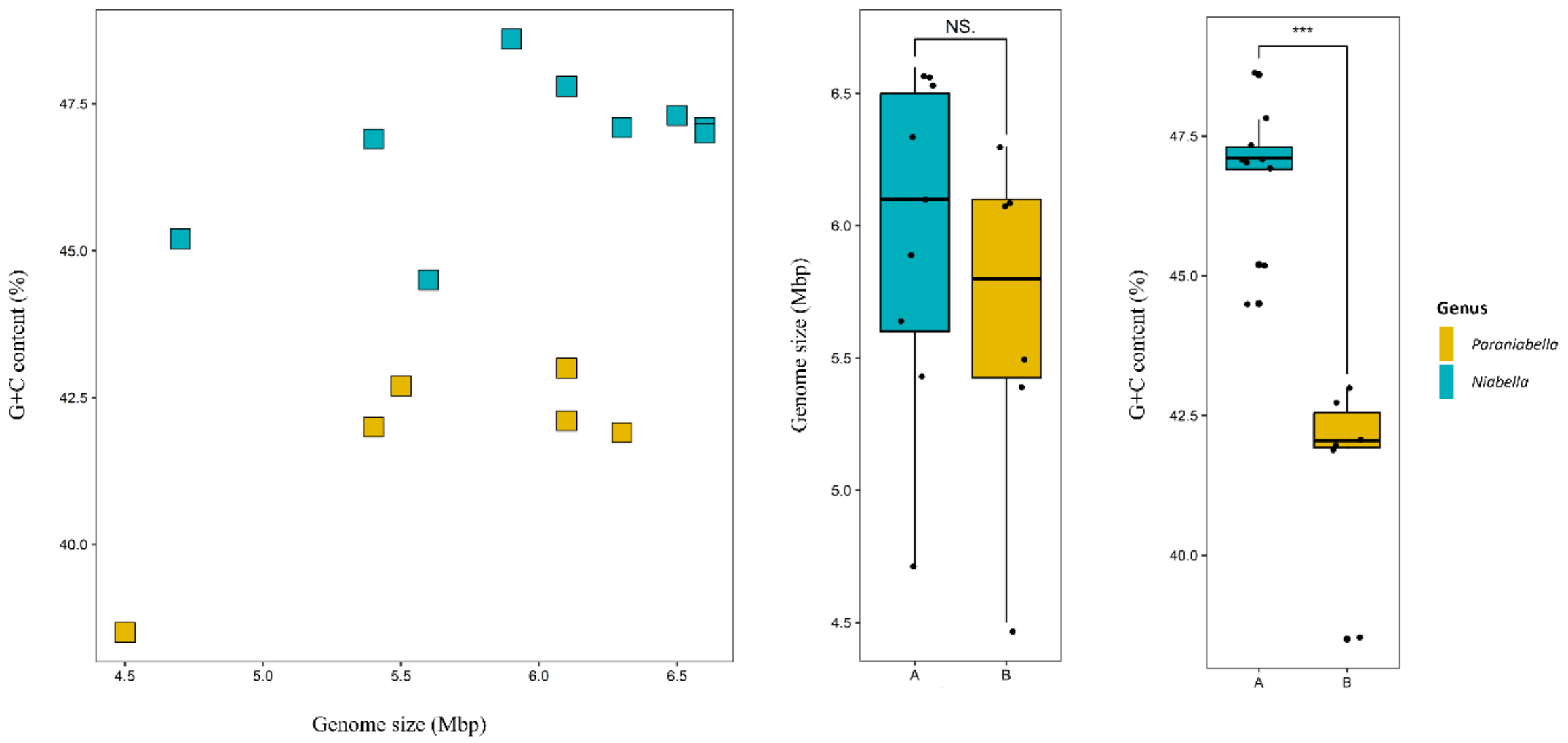

Fig. S6. Scatter and box plots for comparison of genome size (Mbp) and G + C content (%) between the genera *Paraniabella* and *Niabella*. Statistical significance was assessed using a t-test (*NS*, not significant; ***p < 0.001).
