# Supplementary material for: Genome-based classification of Paraniabella aurantiaca gen. nov., sp. nov., isolated from soil and taxonomic reclassification of five species within the genus Niabella
Source: J Microbiol. 2025 Oct 31;63(10):e2505005. doi: 10.71150/jm.2505005 (PMC13576996; doi:10.71150/jm.2505005)
Supplement: Supplementary file 7 [file jm-2505005-Supplementary-Fig-S7.pdf]

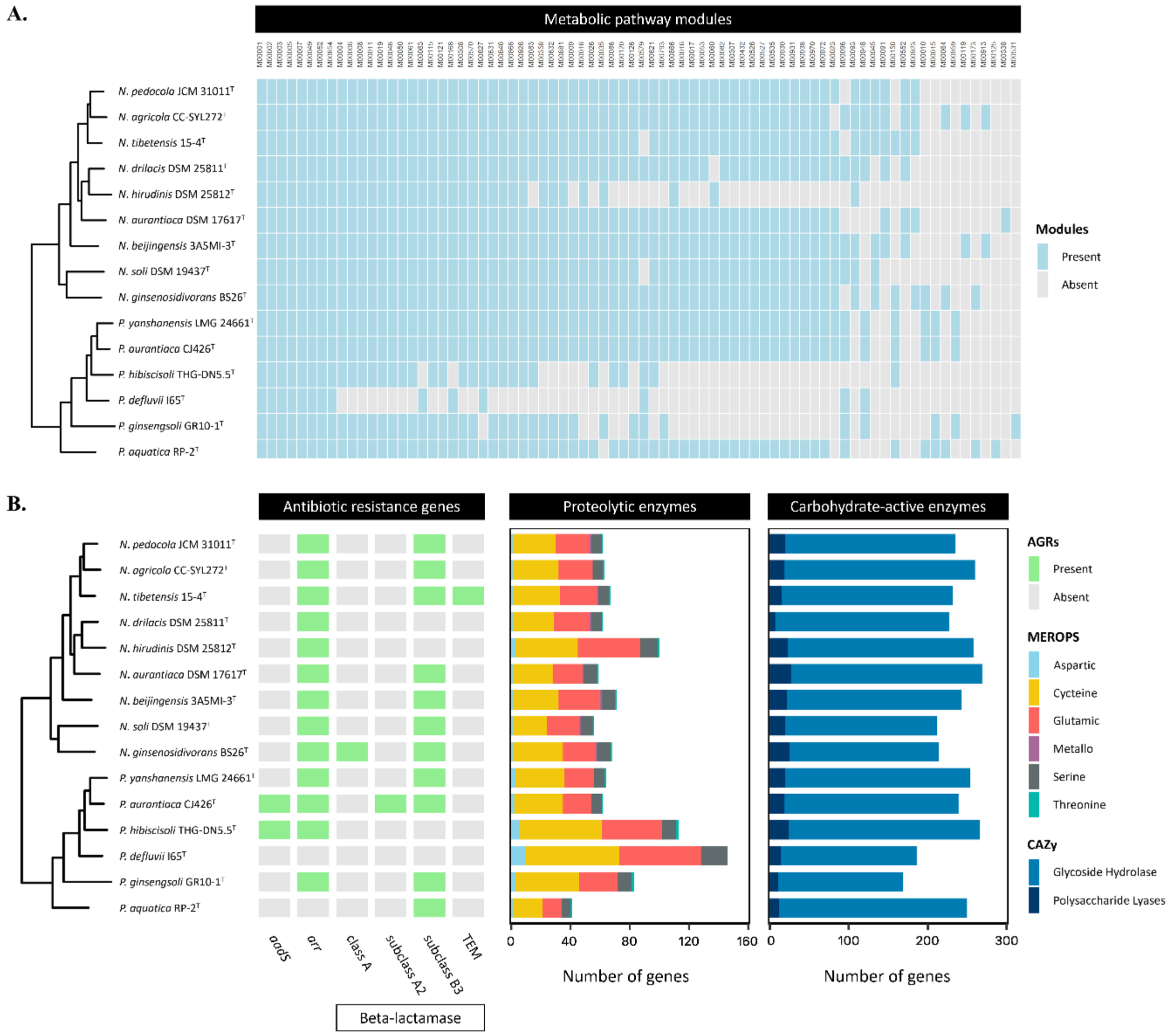

Fig. S7. Functional genomic analysis of strain CJ426^T and related type strains. (A) Heatmap showed the presence or absence of metabolic pathway modules based on KEGG annotations. (B) Comparative analysis of functional genes among the type strains based on various databases. The heatmap showed the presence or absence of antibiotic resistance genes. The Bar plot represents the distribution of proteolytic enzymes and CAZYmes. The dendrogram was a phylogenomic tree based on 81 UBCG.
