# Supplementary material for: Genome-based classification of Paraniabella aurantiaca gen. nov., sp. nov., isolated from soil and taxonomic reclassification of five species within the genus Niabella
Source: J Microbiol. 2025 Oct 31;63(10):e2505005. doi: 10.71150/jm.2505005 (PMC13576996; doi:10.71150/jm.2505005)
Supplement: Supplementary file 8 [file jm-2505005-Supplementary-Fig-S8.pdf]

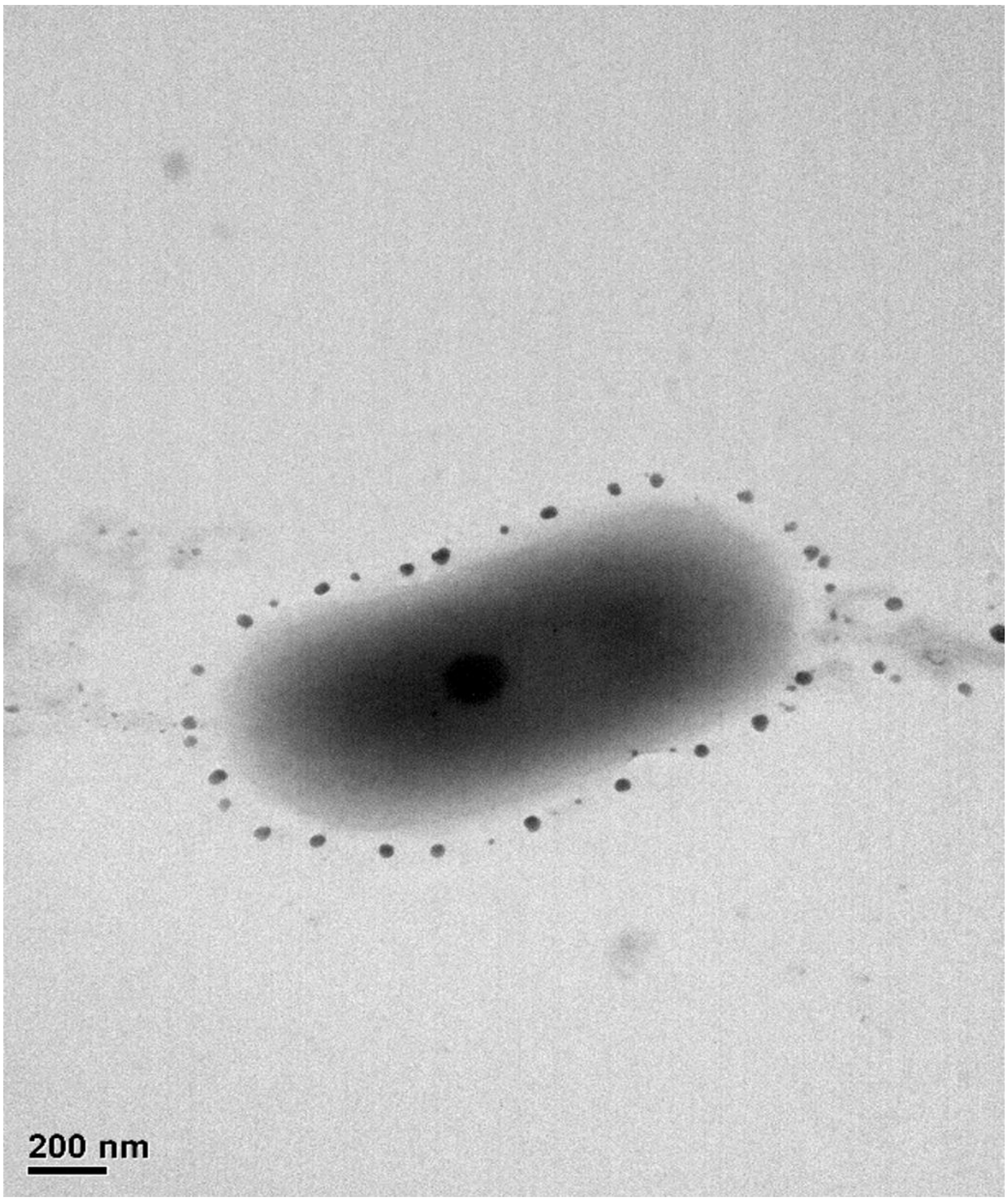

Fig. S8. Transmission electron micrograph (TEM) of strain CJ426^T. Cells were grown on R2A agar for 2 days at 30°C.
