# Supplementary material for: Genome-based classification of Paraniabella aurantiaca gen. nov., sp. nov., isolated from soil and taxonomic reclassification of five species within the genus Niabella
Source: J Microbiol. 2025 Oct 31;63(10):e2505005. doi: 10.71150/jm.2505005 (PMC13576996; doi:10.71150/jm.2505005)
Supplement: Supplementary file 9 [file jm-2505005-Supplementary-Fig-S9.pdf]

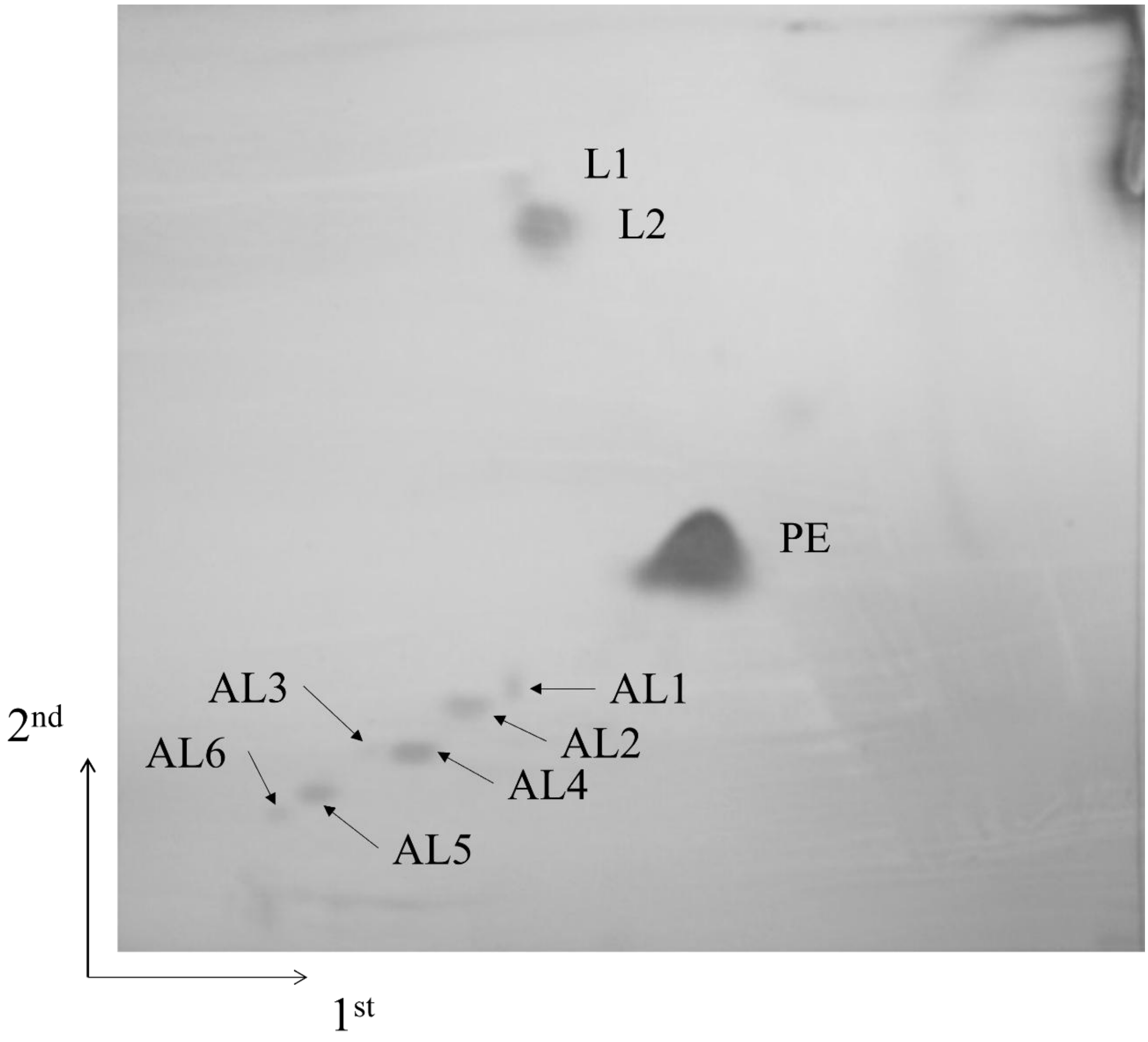

Fig. S9. Two-dimensional thin-layer chromatography of the polar lipids of strain CJ426^T. First dimension, chloroform/methanol/water (65:25:4); second dimension, chloroform/methanol/acetic acid/water (80:12:15:4). Abbreviations: PE, phosphatidylethanolamine; AL, amino lipid; L, unidentified lipid.
