# Supplementary material for: Genome-based classification of Paraniabella aurantiaca gen. nov., sp. nov., isolated from soil and taxonomic reclassification of five species within the genus Niabella
Source: J Microbiol. 2025 Oct 31;63(10):e2505005. doi: 10.71150/jm.2505005 (PMC13576996; doi:10.71150/jm.2505005)
Supplement: Supplementary file 10 [file jm-2505005-Supplementary-Table-S1.pdf]

Table S1. 16S rRNA gene similarity between strain CJ426^T and related type strains

**CJ426^T** vs	**Accession**	**Similarity (%)**
N. agricola CC-SYL272^T	MZ452987	93.23
N. aurantiaca DSM 17617^T	DQ457019	94.31
N. beijingensis 3A5MI-3^T	MZ919349	93.16
N. drilacis DSM 25811^T	KC307772	94.14
N. ginsenosidivorans BS26^T	JQ349050	93.50
N. hirudinis DSM 25812^T	KC307771	94.66
N. pedocola JCM 31011^T	KT032154	94.13
N. soli DSM 19437^T	EF592608	95.52
N. tibetebsis 15-4^T	GU291295	92.34
N. aquatica RP-2^T	KT950742	95.39
N. defluvii I65^T	OM095447	97.77
N. ginsengisoli GR10-1^T	EU616816	96.58
N. hibiscisoli THG-DN5.5^T	KU505213	98.74
N. yanshanensis HAMBI 3031^T	FJ457040	98.11
