# Supplementary material for: Genome-based classification of Paraniabella aurantiaca gen. nov., sp. nov., isolated from soil and taxonomic reclassification of five species within the genus Niabella
Source: J Microbiol. 2025 Oct 31;63(10):e2505005. doi: 10.71150/jm.2505005 (PMC13576996; doi:10.71150/jm.2505005)
Supplement: Supplementary file 11 [file jm-2505005-Supplementary-Table-S10.pdf]

Table S10. Fatty acid composition of strain CJ426^T and related type strains. Strains: 1, CJ426^T; 2, *P. hibiscisoli* THG-DN5.5^T; 3, *P. yanshanensis* HAMBI 3031^T; 4, *P. defluvii* I65^T; 5, *P. ginsengisoli* GR10-1^T; 6, *P. aquatica* RP-2^T; 7, *N. beijingensis* 3A5MI-3^T; 8, *N. soli* DSM 19437^T; 9, *N. tibetensis* 15-4^T; 10, *N. agricola* CC-SYL272^T; 11, *N. pedocola* JCM 31011^T; 12, *N. aurantiaca* DSM 17617^T; 13, *N. drilacis* DSM 25811^T; 14, *N. hirudinis* DSM 25812^T; 15, *N. ginsenosidivorans* BS26^T. -, Fatty acid amounts < 1.0% or not detected. The data for strain CJ426 was obtained from this study, while the data for other strains were taken from published literature.

Fatty acid	1	2	3	4	5	6	7	8	9	10	11	12	13	14	15
**Straight**															
$C_{14:0}$	1.1	-	1.3	-	1.1	-	-	-	-	1.2	-	-	1.4	1.3	-
$C_{16:0}$	5.9	2.9	5.4	4.2	7.1	2.2	5.5	6.8	3.4	4.4	4.3	3.5	3.2	2.9	2.1
$C_{18:0}$	-	-	-	1.6	-	-	-	3.8	-	-	-	-	-	-	-
**Branched**															
iso-$C_{15:0}$	38.2	36.6	36.5	47.1	32.5	40.4	40.6	29.2	41.3	27.9	35.0	33.7	40.8	52.4	42.0
iso-$C_{15:1}$ G*	20.2	26.1	23.0	20.7	22.1	23.4	19.0	18.4	14.9	17.2	24.9	22.3	20.5	16.6	15.9
**Unsaturated**															
$C_{16:1}\ \omega 5c$	1.4	2.2	-	-	2.4	-	-	-	-	-	-	-	-	-	-

$C_{18:1}$ $\omega 9c$	-	-	1.3	-	-	-	-	1.0	-	1.9	-	-	-	-	-
anteiso-$C_{17:1}$ $\omega 9c$	-	-	-	-	1.0	-	-	-	-	-	-	-	-	-	-
**Hydroxy**															
$C_{16:0}$ 3-OH	2.0	1.8	1.9	-	3.1	1.8	2.4	2.2	3.3	4.2	2.0	2.4	3.5	1.9	1.2
iso $C_{15:0}$ 3-OH	2.8	3.0	2.6	2.9	3.0	2.8	1.7	2.2	2.4	3.2	2.8	2.9	-	3.8	2.9
iso $C_{17:0}$ 3-OH	14.0	12.7	12.9	9.9	11.8	13.9	11.4	11.8	13.2	13.8	12.6	15.5	9.2	9.7	16.4
**Summed feature 3**†	7.2	7.2	7.2	8.6	7.8	12.5	7.1	11.1	16.0	13.4	14.4	10.6	10.7	5.2	14.4

*iso-$C_{15:1}$ G should correspond to either iso- $C_{15:1}$ $\omega 6c$ and/or iso- $C_{15:1}$ $\omega 7c$.

†Summed features represent groups of two or three fatty acids that could not be separated by GLC with the MIDI system. Summed feature 3 comprises $C_{16:1}$ $\omega 7c$ and/or $C_{16:1}$ $\omega 6c$.
