# Supplementary material for: Genome-based classification of Paraniabella aurantiaca gen. nov., sp. nov., isolated from soil and taxonomic reclassification of five species within the genus Niabella
Source: J Microbiol. 2025 Oct 31;63(10):e2505005. doi: 10.71150/jm.2505005 (PMC13576996; doi:10.71150/jm.2505005)
Supplement: Supplementary file 12 [file jm-2505005-Supplementary-Table-S2.pdf]

Table S2. Comparison 16S rRNA gene similarity of the type strains within the genus *Niabella*

No.	16S rRNA gene similarity (%)	1	2†	3	4	5	6	7	8	9	10	11	12	13	14
1	*N. agricola* CC-SYL272^{T}	-	95.97	94.19	97.14	93.02	95.96	97.78	94.58	94.96	95.17	94.24	93.99	93.23	93.82
2†	*N. aurantiaca* DSM 17617^{T}	95.97	-	94.88	97.58	93.57	97.19	96.88	95.16	93.82	95.58	94.05	94.79	94.10	93.75
3	*N. beijingensis* 3A5MI-3^{T}	94.19	94.88	-	92.10	93.85	94.30	95.30	95.02	94.25	94.20	94.31	92.60	92.80	93.53
4	*N. drilacis* DSM 25811^{T}	97.14	97.58	92.10	-	93.70	98.02	96.41	95.81	95.88	95.68	94.64	94.88	94.51	94.58
5	*N. ginsenosidivorans* BS26^{T}	93.02	93.57	93.85	93.70	-	93.50	92.71	94.29	91.32	94.12	94.46	93.22	94.05	94.32
6	*N. hirudinis* DSM 25812^{T}	95.96	97.19	94.30	98.02	93.50	-	96.39	95.84	94.30	95.40	94.50	95.09	94.44	94.33
7	*N. pedocola* JCM 31011^{T}	97.78	96.88	95.30	96.41	92.71	96.39	-	94.58	95.53	94.87	93.68	93.97	93.62	93.45
8	*N. soli* DSM 19437^{T}	94.58	95.16	95.02	95.81	94.29	95.84	94.58	-	93.37	95.02	95.73	94.22	95.60	95.48
9	*N. tibetebsis* 15-4^{T}	94.96	94.82	94.25	95.93	91.82	94.87	95.53	93.71	-	92.26	93.02	91.90	91.53	92.73
10	*N. aquatica* RP-2^{T}	95.17	95.58	94.20	95.68	94.12	95.38	94.87	95.02	92.96	-	96.82	96.48	95.44	96.58
11	*N. defluvii* I65^{T}	94.24	94.05	94.31	94.64	94.46	94.50	95.09	94.44	93.02	96.82	-	96.98	97.53	99.56
12	*N. ginsengisoli* GR10-1^{T}	93.99	94.82	92.60	94.88	93.22	95.09	93.97	94.22	91.90	96.48	96.98	-	97.03	97.02
13	*N. hibiscisoli* THG-DN5.5^{T}	93.23	94.06	92.80	94.51	94.05	94.44	93.62	95.60	91.53	95.44	97.53	97.03	-	97.67
14	*N. yanshanensis* HAMBI 3031^{T}	93.82	93.75	93.53	94.58	94.32	94.33	93.45	95.48	92.73	96.58	99.56	97.02	97.67	-

† The Genus type species
