# Supplementary material for: Genome-based classification of Paraniabella aurantiaca gen. nov., sp. nov., isolated from soil and taxonomic reclassification of five species within the genus Niabella
Source: J Microbiol. 2025 Oct 31;63(10):e2505005. doi: 10.71150/jm.2505005 (PMC13576996; doi:10.71150/jm.2505005)
Supplement: Supplementary file 13 [file jm-2505005-Supplementary-Table-S3.pdf]

Table S3. OrthoANI, AAI, POCP and dDDH values between strain CJ426^T and related type strains

**CJ426^T** vs	**OrthoANI**	**AAI**	**POCP**	**dDDH**
N. agricola CC-SYL272^T	69.92	67.53	58.88	20.4
N. aurantiaca DSM 17617^T	69.57	67.69	56.77	19.6
N. beijingensis 3A5MI-3^T	69.45	67.52	57.29	18.8
N. drilacis DSM 25811^T	69.76	67.57	54.69	19.0
N. ginsenosidivorans BS26^T	70.07	67.95	54.10	20.1
N. hirudinis DSM 25812^T	69.68	68.05	55.01	20.4
N. pedocola JCM 31011^T	69.98	67.65	59.08	18.8
N. soli DSM 19437^T	70.04	68.33	56.47	19.7
N. tibetebsis 15-4^T	70.04	67.70	57.12	12.9
N. aquatica RP-2^T	73.59	76.06	60.66	19.6
N. defluvii I65^T	81.47	86.68	68.78	24.3
N. ginsengisoli GR10-1^T	73.93	78.57	62.93	19.9
N. hibiscisoli THG-DN5.5^T	81.40	87.49	75.65	24.3
N. yanshanensis HAMBI 3031^T	84.58	89.95	78.66	28.3
