# Supplementary material for: Genome-based classification of Paraniabella aurantiaca gen. nov., sp. nov., isolated from soil and taxonomic reclassification of five species within the genus Niabella
Source: J Microbiol. 2025 Oct 31;63(10):e2505005. doi: 10.71150/jm.2505005 (PMC13576996; doi:10.71150/jm.2505005)
Supplement: Supplementary file 14 [file jm-2505005-Supplementary-Table-S4.pdf]

Table S4. List of metabolic pathway modules based on KEGG databases

Module	Description
M00001	Glycolysis (Embden-Meyerhof pathway), glucose => pyruvate
M00002	Glycolysis, core module involving three-carbon compounds
M00003	Gluconeogenesis, oxaloacetate => fructose-6P
M00004	Pentose phosphate pathway (Pentose phosphate cycle)
M00005	PRPP biosynthesis, ribose 5P => PRPP
M00006	Pentose phosphate pathway, oxidative phase, glucose 6P => ribulose 5P
M00007	Pentose phosphate pathway, non-oxidative phase, fructose 6P => ribose 5P
M00008	Entner-Doudoroff pathway, glucose-6P => glyceraldehyde-3P + pyruvate
M00009	Citrate cycle (TCA cycle, Krebs cycle)
M00010	Citrate cycle, first carbon oxidation, oxaloacetate => 2-oxoglutarate
M00011	Citrate cycle, second carbon oxidation, 2-oxoglutarate => oxaloacetate
M00015	Proline biosynthesis, glutamate => proline
M00016	Lysine biosynthesis, succinyl-DAP pathway, aspartate => lysine
M00017	Methionine biosynthesis, aspartate => homoserine => methionine
M00018	Threonine biosynthesis, aspartate => homoserine => threonine
M00019	Valine/isoleucine biosynthesis,<br>pyruvate => valine / 2-oxobutanoate => isoleucine
M00023	Tryptophan biosynthesis, chorismate => tryptophan
M00026	Histidine biosynthesis, PRPP => histidine
M00035	Methionine degradation
M00045	Histidine degradation, histidine => N-formiminoglutamate => glutamate
M00048	De novo purine biosynthesis, PRPP + glutamine => IMP

Module	Description
M00049	Adenine ribonucleotide biosynthesis, IMP => ADP,ATP
M00050	Guanine ribonucleotide biosynthesis, IMP => GDP,GTP
M00052	Pyrimidine ribonucleotide biosynthesis, UMP => UDP/UTP,CDP/CTP
M00053	Deoxyribonucleotide biosynthesis, ADP/GDP/CDP/UDP => dATP/dGTP/dCTP/dUTP
M00060	KDO2-lipid A biosynthesis, Raetz pathway, LpxL-LpxM type
M00061	D-Glucuronate degradation, D-glucuronate => pyruvate + D-glyceraldehyde 3P
M00063	CMP-KDO biosynthesis
M00064	ADP-L-glycero-D-manno-heptose biosynthesis
M00082	Fatty acid biosynthesis, initiation
M00083	Fatty acid biosynthesis, elongation
M00086	beta-Oxidation, acyl-CoA synthesis
M00091	Phosphatidylcholine (PC) biosynthesis, PE => PC
M00093	Phosphatidylethanolamine (PE) biosynthesis, PA => PS => PE
M00096	C5 isoprenoid biosynthesis, non-mevalonate pathway
M00115	NAD biosynthesis, aspartate => quinolinate => NAD
M00119	Pantothenate biosynthesis, valine/L-aspartate => pantothenate
M00120	Coenzyme A biosynthesis, pantothenate => CoA
M00121	Heme biosynthesis, plants and bacteria, glutamate => heme
M00125	Riboflavin biosynthesis, plants and bacteria, GTP => riboflavin/FMN/FAD
M00126	Tetrahydrofolate biosynthesis, GTP => THF
M00156	Cytochrome c oxidase, cbb3-type
M00168	CAM (Crassulacean acid metabolism), dark
M00173	Reductive citrate cycle (Arnon-Buchanan cycle)

Module	Description
M00307	Pyruvate oxidation, pyruvate => acetyl-CoA
M00308	Semi-phosphorylative Entner-Doudoroff pathway, gluconate => glycerate-3P
M00338	Cysteine biosynthesis, homocysteine + serine => cysteine
M00358	Coenzyme M biosynthesis
M00432	Leucine biosynthesis, 2-oxoisovalerate => 2-oxoisocaproate
M00526	Lysine biosynthesis, DAP dehydrogenase pathway, aspartate => lysine
M00527	Lysine biosynthesis, DAP aminotransferase pathway, aspartate => lysine
M00531	Assimilatory nitrate reduction, nitrate => ammonia
M00535	Isoleucine biosynthesis, pyruvate => 2-oxobutanoate
M00552	D-galactonate degradation, De Ley-Doudoroff pathway, D-galactonate => glycerate-3P
M00570	Isoleucine biosynthesis, threonine => 2-oxobutanoate => isoleucine
M00579	Phosphate acetyltransferase-acetate kinase pathway, acetyl-CoA => acetate
M00621	Glycine cleavage system
M00627	beta-Lactam resistance, Bla system
M00631	D-Galacturonate degradation (bacteria), D-galacturonate => pyruvate + D-glyceraldehyde 3P
M00632	Galactose degradation, Leloir pathway, galactose => alpha-D-glucose-1P
M00793	dTDP-L-rhamnose biosynthesis
M00846	Siroheme biosynthesis, glutamyl-tRNA => siroheme
M00854	Glycogen biosynthesis, glucose-1P => glycogen/starch
M00866	KDO2-lipid A biosynthesis, Raetz pathway, non-LpxL-LpxM type
M00868	Heme biosynthesis, animals and fungi, glycine => heme
M00881	Lipoic acid biosynthesis, plants and bacteria, octanoyl-ACP => dihydrolipoyl-E2/H
M00913	Pantothenate biosynthesis, 2-oxoisovalerate/spermine => pantothenate

Module	Description
M00923	UDP-L-FucNAm biosynthesis
M00926	Heme biosynthesis, bacteria, glutamyl-tRNA => coproporphyrin III => heme
M00930	Menaquinone biosynthesis, futalosine pathway
M00931	Menaquinone biosynthesis, modified futalosine pathway
M00938	Pyrimidine deoxyribonucleotide biosynthesis, UDP => dTTP
M00948	Hydroxyproline degradation, trans-4-hydroxy-L-proline => 2-oxoglutarate
M00959	Guanine ribonucleotide degradation, GMP => Urate
M00970	Proline degradation, proline => glutamate
M00972	Proline metabolism
