# Supplementary material for: Genome-based classification of Paraniabella aurantiaca gen. nov., sp. nov., isolated from soil and taxonomic reclassification of five species within the genus Niabella
Source: J Microbiol. 2025 Oct 31;63(10):e2505005. doi: 10.71150/jm.2505005 (PMC13576996; doi:10.71150/jm.2505005)
Supplement: Supplementary file 15 [file jm-2505005-Supplementary-Table-S5.pdf]

Table S5. Antimicrobial susceptibility (inhibition zone diameter; mm) of *Paraniabella aurantiaca* CJ426^T

Antibiotics	CJ426^T	Susceptible/Resistant*
Amoxicillin (10 μg)	6.0	R
Cephalexin (30 μg)	42.9	S
Chloramphenicol (10 μg)	11.1	R
Ciprofloxacin (5 μg)	32.9	S
Clindamycin (2 μg)	30.3	S
Colistin (10 μg)	6.0	R
Erythromycin (10 μg)	6.0	R
Fosfomycin (200 μg)	6.0	R
Gentamicin (10 μg)	16.9	I
Linezolid (10 μg)	10.5	R
Meropenem (10 μg)	11.8	R
Rifampicin (5 μg)	9.0	R
Streptomycin (5 μg)	6.0	R
Sulfamethoxazole (50 μg)	19.7	S
Tetracycline (30 μg)	11.7	I
Trimethoprim (5 μg)	6.0	R
Tylosin (30 μg)	27.5	S
Vancomycin (5 μg)	6.0	R

*Susceptibility results were recorded as susceptible (S), intermediate (I) or resistant (R) based on breakpoints for *Enterobacteriaceae* and *Staphylococcus* spp. according to the CLSI supplement M100.
