# Supplementary material for: Genome-based classification of Paraniabella aurantiaca gen. nov., sp. nov., isolated from soil and taxonomic reclassification of five species within the genus Niabella
Source: J Microbiol. 2025 Oct 31;63(10):e2505005. doi: 10.71150/jm.2505005 (PMC13576996; doi:10.71150/jm.2505005)
Supplement: Supplementary file 16 [file jm-2505005-Supplementary-Table-S6.pdf]

Table S6. The number of predicted family domains of carbohydrate-active enzymes based on the CAZy database for CJ426^T and related type strains.

Strains	Glycoside hydrolase	Polysaccharide lyases
N. agricola CC-SYL272^T	241	19
N. aurantiaca DSM 17617^T	242	27
N. beijingensis 3A5MI-3^T	221	22
N. drilacis DSM 25811^T	220	7
N. ginsenosidivorans BS26^T	189	25
N. hirudinis DSM 25812^T	235	23
N. pedocola JCM 31011^T	215	20
N. soli DSM 19437^T	192	20
N. tibetebsis 15-4^T	217	15
P. aquatica RP-2^T	238	12
P. aurantiaca CJ423^T	220	19
P. defluvii I65^T	172	14
P. ginsengisoli GR10-1^T	158	11
P. hibiscisoli THG-DN5.5^T	242	24
P. yanshanensis HAMBI 3031^T	234	20
