# Supplementary material for: Genome-based classification of Paraniabella aurantiaca gen. nov., sp. nov., isolated from soil and taxonomic reclassification of five species within the genus Niabella
Source: J Microbiol. 2025 Oct 31;63(10):e2505005. doi: 10.71150/jm.2505005 (PMC13576996; doi:10.71150/jm.2505005)
Supplement: Supplementary file 17 [file jm-2505005-Supplementary-Table-S7.pdf]

Table S7. The number of predicted family domains of proteolytic enzymes based on the MEROPS database for CJ426^T and related type strains.

-, not detected

Strains	Aspartic peptidase	Cysteine peptidase	Glutamic peptidase	Metallo peptidase	Serine peptidase	Threonine peptidase	Peptidase inhibitors
N. agricola CC-SYL272^T	1	7	-	23	31	1	5
N. aurantiaca DSM 17617^T	1	6	-	13	20	1	-
N. beijingensis 3A5MI-3^T	1	9	1	20	27	1	1
N. drilacis DSM 25811^T	1	9	1	28	31	1	1
N. ginsenosidivorans BS26^T	-	18	-	55	63	10	14
N. hirudinis DSM 25812^T	1	7	1	24	28	1	1
N. pedocola JCM 31011^T	2	9	-	26	43	3	3
N. soli DSM 19437^T	1	9	1	22	34	1	1
N. tibetebsis 15-4^T	2	9	-	41	55	6	7
P. aquatica RP-2^T	1	12	-	42	42	3	8
P. aurantiaca CJ423^T	1	7	1	23	29	1	1
P. defluvii I65^T	1	8	1	22	23	1	1
P. ginsengisoli GR10-1^T	1	7	-	19	33	2	-
P. hibiscisoli THG-DN5.5^T	1	7	1	25	32	1	2
P. yanshanensis HAMBI 3031^T	1	7	-	20	33	3	-
