# Supplementary material for: Genome-based classification of Paraniabella aurantiaca gen. nov., sp. nov., isolated from soil and taxonomic reclassification of five species within the genus Niabella
Source: J Microbiol. 2025 Oct 31;63(10):e2505005. doi: 10.71150/jm.2505005 (PMC13576996; doi:10.71150/jm.2505005)
Supplement: Supplementary file 18 [file jm-2505005-Supplementary-Table-S8.pdf]

Table S8. The information of biosynthetic gene clusters in strain CJ426[T]

Region	Type	From	To	Most similar known cluster	Similarity (%)
1.1	Terpene	1,321,848	1,342,861	-	
1.2	Aryl polyene	1,350,009	1,423,621	Flexirubin	44
1.3	Aryl polyene, Resorcinol	1,678,478	1,743,750	Flexirubin	25
1.4	Lasso peptide	2,367,846	2,390,456	TP-1161	8
1.5	NRPS-like, T1PKS, NRPS	3,343,950	3,394,650	-	
2.1	Terpene	1,772,259	1,793,095	Carotenoid	28
3.1	T3PKS	460,032	501,126	-	
