# Supplementary material for: Genome-based classification of Paraniabella aurantiaca gen. nov., sp. nov., isolated from soil and taxonomic reclassification of five species within the genus Niabella
Source: J Microbiol. 2025 Oct 31;63(10):e2505005. doi: 10.71150/jm.2505005 (PMC13576996; doi:10.71150/jm.2505005)
Supplement: Supplementary file 19 [file jm-2505005-Supplementary-Table-S9.pdf]

Table S9. Characteristics of strain CJ426^T and the closely related type strains within the genera *Niabella* and *Paraniabella*. Strains: 1, CJ426^T; 2, *P. hibiscisoli* THG-DN5.5^T; 3, *P. yanshanensis* HAMBI 3031^T; 4, *P. defluvii* I65^T; 5, *P. ginsengisoli* GR10-1^T; 6, *P. aquatica* RP-2^T; 7, *N. beijingensis* 3A5MI-3^T; 8, *N. soli* DSM 19437^T; 9, *N. tibetensis* 15-4^T; 10, *N. agricola* CC-SYL272^T; 11, *N. pedocola* JCM 31011^T; 12, *N. aurantiaca* DSM 17617^T; 13, *N. drilacis* DSM 25811^T; 14, *N. hirudinis* DSM 25812^T; 15, *N. ginsenosidivorans* BS26^T. The data for strain CJ426 was obtained from this study, while the data for other strains were taken from published literature. +, Positive; w, weakly positive; -, negative; ND, data not available

Characteristic	1	2	3	4	5	6	7	8	9	10	11	12	13	14	15
**Isolation source**	Soil	Soil	Rhizo-sphere	Influent water of WWTP	Soil	Lake water	Wetland	Soil	Desert Soil	Paddy soil	Soil	Soil	Medicinal leeches	Medicinal leeches	Compost
**Growth**															
Temperature (°C)	4–37	4–40	28	20–45	5–35	10–37	15–45	15–35	15–34	20–40	15–40	10–35	20–30	20–30	18–42
pH	5–12	ND	6–10	5–10	6–8	5–9	5–9	5–8	5–8	6–8	55–10.5	5–8	4.5–10.5	4.5–8.5	4.5–10.0
NaCl (%, w/v)	0–2	0–2	0–1	0–2	0–2	0.5–1.5	0–2	0–1	0–1	0–2	0–2	0–3	0–1	0–4	0–3
**Fermentation of**															
Glucose	-	-	+	-	-	ND	ND	-	-	+	-	-	-	-	-
**Indole production**	+	-	ND	w	-	-	-	-	+	-	-	+	ND	ND	-
**Hydrolysis of**															
Casein	+	+	ND	+	-	-	ND	-	+	ND	+	+	ND	ND	-
Starch	+	+	+	+	-	-	+	-	-	ND	-	-	ND	ND	-
CM-Cellulose	+	-	-	-	-	-	w	-	-	ND	ND	-	ND	ND	-

Esculin	+	+	ND	+	+	-	+	+	+	ND	+	+	+	+	+
Gelatin	+	+	-	w	-	-	+	-	-	+	-	-	ND	ND	-
**Enzyme activity**															
Catalase	+	+	+	+	+	+	-	+	+	+	-	+	-	-	+
Oxidase	-	+	+	+	-	-	+	+	+	+	+	-	+	+	+
β-Galactosidase	+	+	ND	w	+	-	+	+	+	+	+	+	ND	ND	+
Urease	-	-	+	-	-	-	-	-	-	-	-	-	ND	ND	+
Arginine dihydrolase	+	-	ND	-	-	-	-	-	-	ND	-	-	ND	ND	+
**Assimilation of**															
D-Glucose	+	+	+	+	+	+	+	+	+	+	+	+	-	-	+
L-Arabinose	+	+	+	-	+	+	+	+	+	+	+	+	+	+	+
D-Mannose	+	+	+	+	+	+	+	+	+	+	+	+	+	+	+
D-Mannitol	-	-	-	-	-	-	-	+	-	-	-	-	-	-	+
D-Maltose	+	+	+	+	+	+	+	+	+	+	+	+	+	-	+
Potassium gluconate	-	w	ND	-	-	+	-	+	-	-	-	-	-	w	+
Malic acid	-	-	ND	-	-	+	-	-	-	-	-	-	-	-	-
